# RAAS antagonists dampen the SARS-CoV-2 infection in *ex-vivo* cultured human precision-cut lung slices

**DOI:** 10.1186/s12931-025-03463-8

**Published:** 2026-01-13

**Authors:** Poornima Mahavadi, Martina Korfei, Christin Müller-Ruttloff, Clemens Ruppert, Ekaterina Krauss, Peter Dorfmüller, Stefan Gattenloehner, Stefanie Dimmeler, Elie El Agha, Saverio Bellusci, Susanne Herold, Biruta Witte, John Ziebuhr, Andreas Guenther

**Affiliations:** 1 Department of Internal Medicine II, Center for Interstitial and Rare Lung Diseases, Biomedical Research Center Seltersberg (BFS), Justus Liebig University Giessen, Giessen, D-35392 Germany; 2 Universities of Giessen and Marburg Lung Center (UGMLC), Member of the German Center for Lung Research (DZL), Justus Liebig University Giessen, Giessen, Germany; 3 Institute of Medical Virology, Justus Liebig University Giessen, Giessen, D-35392 Germany; 4 Member of German Center for Infection Research (DZIF), Giessen, Germany; 5 European IPF Registry and Biobank, Giessen, Germany; 6 Institute of Pathology, University Hospital Giessen, Justus Liebig University Giessen, Giessen, D-35392 Germany; 7 Institute for Cardiovascular Regeneration, Goethe University Frankfurt, Frankfurt, D- 60590 Germany; 8 Member of the Cardiopulmonary Institute (CPI), Giessen, Germany; 9 Institute for Lung Health (ILH), Giessen, D-35392 Germany; 10 Department of Medicine V, Infectious Diseases and Infection Control, Justus- Liebig University Giessen, Giessen, D-35392 Germany; 11 Department of General and Thoracic Surgery, University Hospital Giessen, Giessen, D-35392 Germany; 12 Lung Clinic, Evangelisches Krankenhaus Mittelhessen, Giessen, D-35398 Germany; 13 Center for Interstitial and Rare Lung Diseases, Department of Internal Medicine, Justus Liebig University Giessen, Klinikstrasse 36, Giessen, D-35392 Germany

**Keywords:** Coronavirus disease 2019 (COVID-19), Acute respiratory distress syndrome (ARDS), Cytokine, Angiotensin-converting enzyme 1 inhibitor (ACEi), Angiotensin-II type 1 receptor (AT_1_R) blocker (ARB)

## Abstract

**Background:**

While the renin-angiotensin-aldosterone system (RAAS) is critically involved in pathomechanisms related to SARS-CoV-2 infection, the role of ongoing therapy with angiotensin-converting enzyme 1 inhibitors (ACEi) or Angiotensin-II type 1 receptor (AT_1_R) blockers (ARB) is much less clear. We evaluated the effects of the ACEi enalapril (ENA) and the ARB losartan (LOS) on SARS-CoV-2 infection in human ex vivo-cultured, precision-cut lung slices (PCLS) obtained from normal human lung tissue.

**Methods:**

PCLS were pre-treated for 5d with vehicle, LOS or ENA (300 µM), followed by mock infection or infection with SARS-CoV-2 and incubation with vehicle, LOS or ENA for 1d or 2d. Thereafter, PCLS were harvested for analysis of viral replication, inflammatory responses, endoplasmic reticulum (ER) stress and apoptosis pathways.

**Results:**

Both LOS and ENA significantly reduced viral replication in PCLS, with ENA being more potent. LOS was more efficient than ENA in reducing the expression of *IL1B*, *CCL2*, *CXCL2* and *TNFA*, but not of *IL6*, whereas ENA preferentially caused a reduction of *IL6* and *CCL2* in SARS-CoV-2-infected PCLS. Further, ENA, but not LOS, significantly decreased the expression of viral entry factors, ACE2 and transmembrane serine protease 2 (TMPRSS2), in infected PCLS. Importantly, LOS or ENA did not exert cytotoxic effects.

**Conclusions:**

RAAS-antagonizing drugs do not seem to exert detrimental effects during SARS-CoV-2 infection. In opposite, in an ex-vivo model of human PCLS, such treatment was found to dampen SARS-CoV-2 infection and consecutive inflammation.

**Supplementary Information:**

The online version contains supplementary material available at 10.1186/s12931-025-03463-8.

## Background

The coronavirus disease 2019 (COVID-19) pandemic, caused by the severe acute respiratory syndrome coronavirus 2 (SARS-CoV-2), has evolved to one of the biggest threats of our times. SARS-CoV-2 infection can induce mild or moderate disease with no need of hospitalization [1]. However, in the elderly [2] or in individuals with pre-existing lung disease, immunosuppressive disorders, hypertension, cardiovascular disease (CVD) and diabetes mellitus [1, 3, 4], severe illness may evolve, with pneumonia, lung injury or acute respiratory distress syndrome (ARDS), in combination with a “cytokine storm” [1–3], causing high morbidity and mortality (up to 40% in these cases).

SARS-CoV-2 enters the epithelial cells of the respiratory tract via the primary receptor angiotensin-converting enzyme 2 (ACE2) [4–8]. ACE2 is also known as a component of the renin-angiotensin-aldosterone system (RAAS) which regulates blood pressure, fluid and electrolyte balance, as well as systemic vascular resistance [9, 10]. Whereas the related ACE1 converts angiotensin-I (Ang-I) to its active form Ang-II, a vasoconstrictor with proliferative/profibrotic effects, ACE2 counterbalances this mechanism by targeting Ang-II and converting it to Ang 1–7, which then acts as a beneficial vasodilator. Ang 1–7 is considered as a cytoprotective peptide in RAAS with anti-oxidant, anti-inflammatory, anti-proliferative, anti-fibrotic, potent vasodilatory, and anti-thrombotic properties, which are exerted by activating the Mas-receptor [9, 10].

ACE2 is expressed in multiple organs, such as kidney, intestinal tract, brain, lung, blood vessels and heart [11]. In the lung, ACE2 is expressed in secretory bronchial cells of the proximal airways and in type-I and type-II alveolar epithelial cells (AECI/AECII) of the distal airspaces [11, 12]. Upon SARS-CoV-2 infection, the spike (S) protein of SARS-CoV-2 attaches to the ACE2 protein on the cell surface of target cells, is then cleaved by transmembrane serine protease 2 (TMPRSS2), which induces a conformational change in the S protein that activates fusion activity, thereby triggering fusion of the coronavirus envelope with the plasma membrane and resulting in virus entry [7, 8, 13, 14]. Mechanisms of SARS-CoV-2 entry independent of ACE2, such as via CD147-receptor [8], have also been reported.

In the past, it had been speculated that increased ACE2 levels may contribute to increased susceptibility to SARS-CoV-2 infection [15]. In addition, several experimental studies demonstrated an increase of ACE2 expression in the heart due to ACE inhibitors (ACEi) and Angiotensin-II type 1 receptor (AT_1_R) blockers (ARB), which are widely used as antihypertensive drugs [16, 17]. Because overexpression of human ACE2 was shown to enhance disease severity in mice infected with SARS-CoV [11], it has been speculated that the use of ACEi and ARBs could increase SARS-CoV-2 replication in hypertensive patients [12, 18]. In this context, it is worth mentioning that administration of recombinant ACE2 has previously been shown to have lung-protective effects in acid- or sepsis-induced ARDS in mice [19] and that SARS-CoV-2 downregulates ACE2 through lysosomal degradation and blocking this process has been suggested to be a promising therapeutic strategy [20].

To resolve these partially contradictory findings, we analyzed the effects of the ACEi enalapril (ENA) and the ARB losartan (LOS) on SARS-CoV-2 infection in ex vivo-cultured, human precision-cut lung slices (PCLS). In contrast to previous reports, we obtained evidence for beneficial effects of such treatment, both in view of viral replication as well in view of the inflammatory responses (“cytokine storm”).

## Methods

### Human lung tissue Preparation and generation of high precision-cut lung slices (PCLS)

Peripheral lung tissue samples with normal histology were obtained from unaffected lung areas of 5 patients with lung cancer (controls, mean age ± SD: 58.80 ± 8.76 years; 3 females, 2 males) who underwent video-assisted thoracic surgery (VATS) for lung cancer resection. There were no documented use of steroids or immunosuppressants at the time of VATS (Supplementary Table S1). In addition, subpleural normal lung tissue samples were obtained from explanted lungs of two male organ donors (58 and 30 years). Further, peripheral lung tissue of one explanted idiopathic pulmonary fibrosis (IPF)-lung (male, 59 years old) was also used in this study. All lung tissue samples were collected and provided by the European IPF registry (eurIPFreg) and the UGMLC Giessen Biobank (member of the DZL-Platform Biobanking). The study protocol was approved by the Ethics Committee of the Justus Liebig University Giessen (No. 111/08 and 58/15).

300 μm-thick PCLS of human lung tissues were generated by agarose filling followed by slicing with a vibratome (ThermoFisher Scientific), and cultured in RPMI medium supplemented with 2% fetal bovine serum (Sigma).

### Drug treatments, viral infection and tissue-based analyses

PCLS were pre-treated for 5d with 0.33% DMSO (vehicle), 300 µM losartan (LOS, Sigma) or 300 µM enalaprilat dihydrate (ENA, Selleckchem), in line with the previously published studies [21] followed by infection of PCLS with mock/vehicle or 1 × 10^5^ plaque-forming units (pfu) of SARS-CoV-2 at 33 °C on day 6 and incubation for 1d or 2d in the (continued) presence or absence of LOS and ENA. PCLS from each condition were harvested and used for subsequent mRNA- (*n* = 3), protein- (*n* = 4–5), and immunohistochemistry (IHC) analyses (*n* = 3) at 1 and 2 days post infection (d.p.i.).

A detailed description of the experimental procedures including quantitative Real-Time Polymerase Chain Reaction (qRT-PCR), list of primers (Table S2), immunoblotting and IHC, are available in the online supplement.

### Statistics

All data were analyzed by GraphPad Prism 8.0.1 software, and are presented as means ± SD. *n* is described in the figure legends and refers to the number of independent treatments. Statistical significance of differences between ≥ 3 groups was evaluated using parametric one-way ANOVA followed by Bonferroni´s multiple comparisons test, or by non-parametric Kruskal-Wallis test followed by Dunn´s multiple comparisons test, where appropriate. Significance level is indicated by *p < 0.05, **p < 0.01, ***p < 0.001.

## Results

**Fig. 1:**
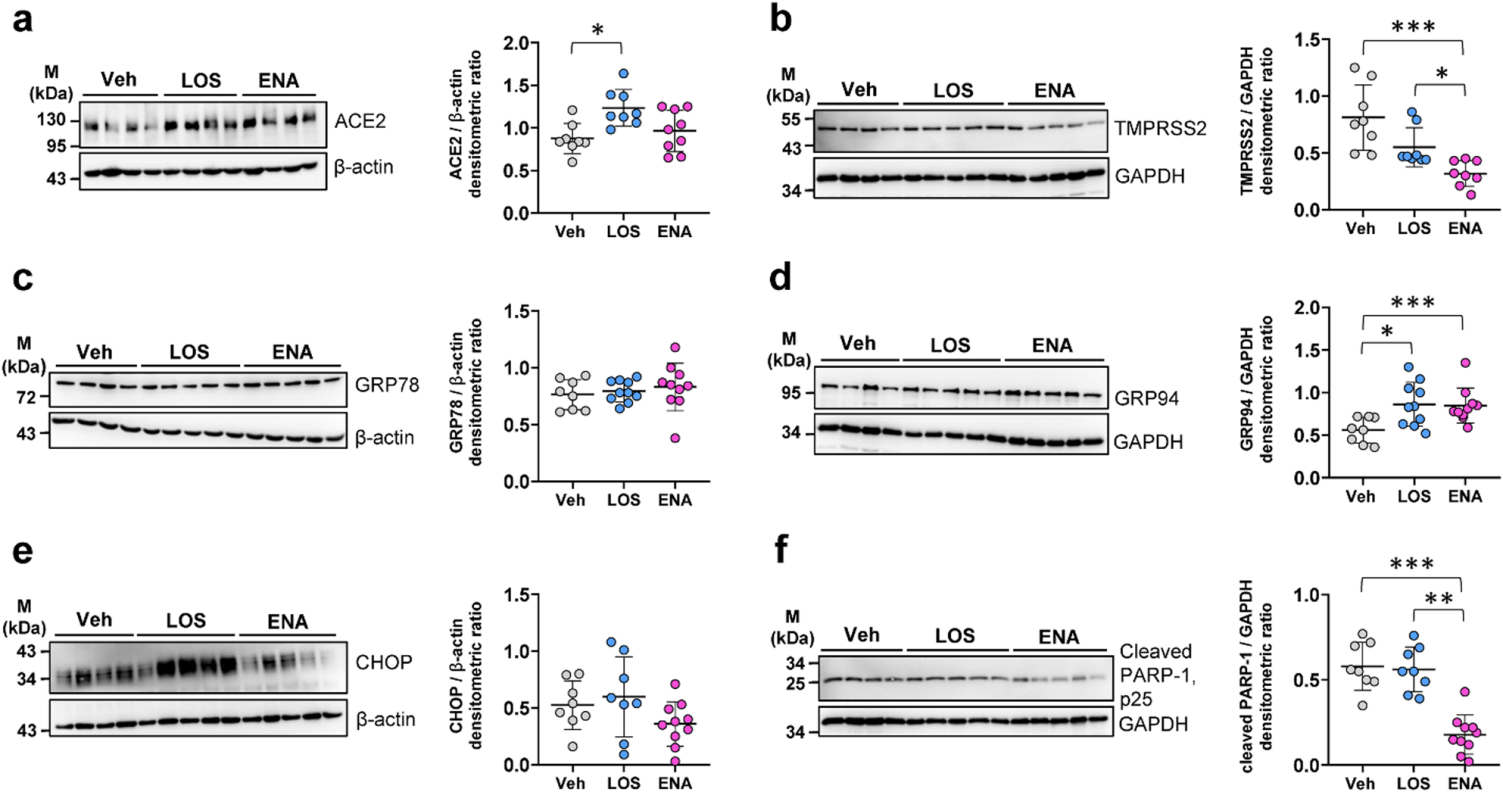
Protein expression of ACE2, TMPRSS2, ER stress markers and cleaved PARP-1 in cultured precision-cut lung slices (PCLS) in response to treatment with losartan or enalapril. Cultured PCLS were incubated for 6d with vehicle [Veh, 0,33% (v/v) DMSO], losartan (LOS, 300 µM) or enalapril (ENA, 300 µM), followed by immunoblot analyses of harvested PCLS. Quantitative immunoblotting for (**a**) ACE2, (**b**) TMPRSS2, (**c**) GRP78, (**d**) GRP94, (**e**) CHOP, and (**f**) cleaved PARP-1 p25, GAPDH or β-actin served as loading control. Data are presented as means ± SD (*n* = 4–5 distinct treatments, from two experiments). ***p < 0.001, **p < 0.01, *p < 0.05, by Dunn´s multiple comparisons test

We first examined the effects of RAAS-inhibiting drugs losartan (LOS) and enalapril (ENA) on the levels of SARS-CoV-2 entry factors and ER stress markers in (non-infected) ex vivo-cultured human PCLS. Comparative immunoblot analysis revealed a significant upregulation of ACE2 protein levels in response to treatment with LOS as compared to treatment with the vehicle (Fig. 1a), whereas *ACE2* mRNA levels remained unchanged (Figure S1a). In contrast, protein levels for TMPRSS2 were markedly downregulated in response to LOS / ENA vs. vehicle (Fig. 1b), which was also evident at mRNA level (Figure S1b).

With regard to ER stress markers, LOS and ENA did not significantly alter protein levels of 78 kDa glucose-regulated protein (GRP78) and C/EBP-homologous protein (CHOP) (Fig. 1c and e), but significantly increased those of GRP94 vs. vehicle (Fig. 1d). Importantly, LOS or ENA treatment did not induce apoptosis, and ENA actually reduced endogenous levels of cleaved poly [ADP-ribose] polymerase 1 (PARP-1), an apoptosis-indicating protein (Fig. 1f). Signs of cell cycle arrest were also not observed in the presence of both RAAS-inhibiting drugs, as shown by qRT-PCR for *CIP1*/p21 (Figure S1g).

**Fig. 2:**
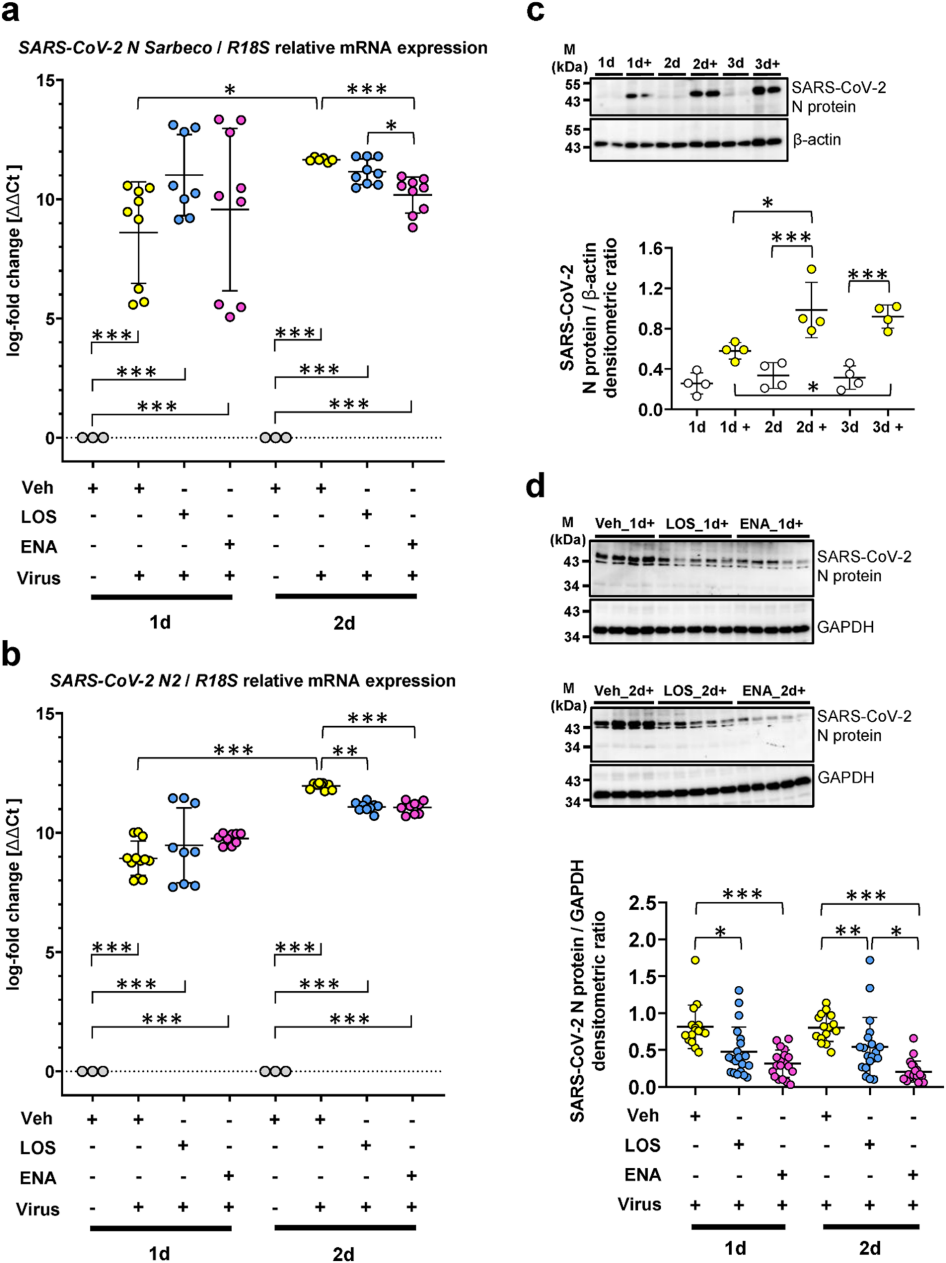
Effects of losartan and enalapril on SARS-CoV-2 infection in human precision-cut lung slices. Cultured human PCLS were pretreated for 5d with vehicle [Veh, 0,33% (v/v) DMSO], losartan (LOS, 300 µM) or enalapril (ENA, 300 µM), followed by infection of PCLS with vehicle or SARS-CoV-2 for 1d or 2d in the presence or absence of losartan and enalapril. PCLS were harvested for RNA isolation, and analyzed by quantitative RT-PCR for SARS-CoV-2 infection using specific primers given in Table S3 (**a**) or published by Qiagen (**b**). *R18S* served as reference gene. The log_2_ fold-changes are given by the ΔΔCt values, where ΔΔCt = ΔCt[treatment] - ΔCt[untreated (Veh_1d or Veh_2d)]. Data are representative for *n* = 3 distinct treatments, with *n* = 3 technical replicates. Data are expressed as means ± SD. ***p < 0.001, **p < 0.01, *p < 0.05, by Bonferroni´s multiple comparisons test. (**c**) Establishment of a specific antibody against SARS-CoV-2 infection. Human PCLS were treated with vehicle or SARS-CoV-2 (+) for 1d, 2d and 3d, followed by quantitative immunoblot analysis for SARS-CoV-2 N protein. Data are representative for *n* = 2 treatments per timepoint and 2 technical replicates. Data are expressed as means ± SD. ***p < 0.001, *p < 0.05, by Bonferroni´s multiple comparisons test. (**d**) Representative and quantitative immunoblot-analysis for SARS-CoV-2 N protein in virus-infected PCLS in the absence (Veh) or presence of losartan (LOS) and enalapril (ENA) at 1 or 2 d.p.i. Data are representative for *n* = 4 independent experimental setups with four different normal human lungs, with *n* = 4–5 distinct treatments per experiment. Data are expressed as means ± SD. ***p < 0.001, **p < 0.01, *p < 0.05 by Dunn´s multiple comparisons test

Next, PCLS generated from normal human lung tissue were pretreated for 5d with vehicle, LOS or ENA, followed by mock infection or infection of PCLS with SARS-CoV-2 for 1d or 2d in the presence or absence of LOS and ENA. RNA was isolated from PCLS and SARS-CoV-2 RNA was measured using primers specific for the viral *N* gene (Table S3; Fig. 2a and b, Figure S7). RT-qPCR analyses using the two sets of SARS-CoV-2 *N* gene-specific primers indicated high concentrations of viral RNA at 1 d.p.i., which further increased at 2 d.p.i. in PCLS in the absence of drugs (Fig. 2a and b). We also found that, in PCLS treated with LOS or ENA, viral RNA concentrations were slightly reduced at 2 d.p.i. as compared to vehicle-treated PCLS (Fig. 2a and b). Immunoblot analysis using SARS-CoV-2 N protein-specific antibodies revealed an accumulation of the N protein in PCLS at 1 d.p.i., which further increased over time at 2 and 3 d.p.i. (Fig. 2c), confirming efficient SARS-CoV-2 replication in lung cells. As shown in Fig. 2D, treatment of the ex vivo-cultured PCLS cells with LOS and ENA, respectively, significantly decreased the accumulation of viral N protein, with ENA causing a more pronounced reduction of viral N protein levels (Fig. 2d).

**Fig. 3:**
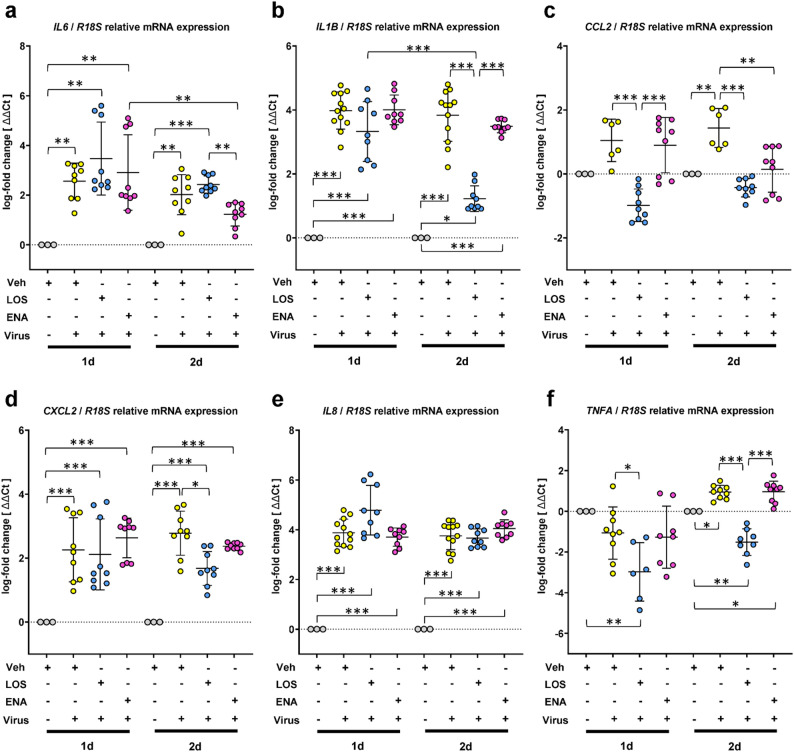
Effects of losartan and enalapril on cytokine storm in SARS-CoV-2-infected human precision-cut lung slices. Analysis of gene expression for *IL6* (**a**), *IL1B* (**b**), *CCL2* (**c**), *CXCL2* (**d**) *IL8* (**e**) and *TNFA* (**f**) in response to SARS-CoV-2 infection in the absence or presence of losartan or enalapril treatment. Cultured human PCLS were pretreated for 5 days with vehicle [Veh, 0,33% (v/v) DMSO], losartan (LOS, 300 µM) or enalapril (ENA, 300 µM), followed by infection of PCLS with vehicle or SARS-CoV-2 for 1d or 2d in the presence or absence of losartan and enalapril. PCLS were harvested for RNA isolation, and analyzed by qRT-PCR for indicated genes. *R18S* served as reference gene. The log_2_ fold-changes are given by the ΔΔCt values, where ΔΔCt = ΔCt[treatment] - ΔCt[untreated (Veh_1d or Veh_2d)]. Data are representative for *n* = 3 distinct treatments, with *n* = 3 technical replicates. Data are expressed as means ± SD. ***p < 0.001, **p < 0.01, *p < 0.05, by Bonferroni´s multiple comparisons test

**Fig. 4:**
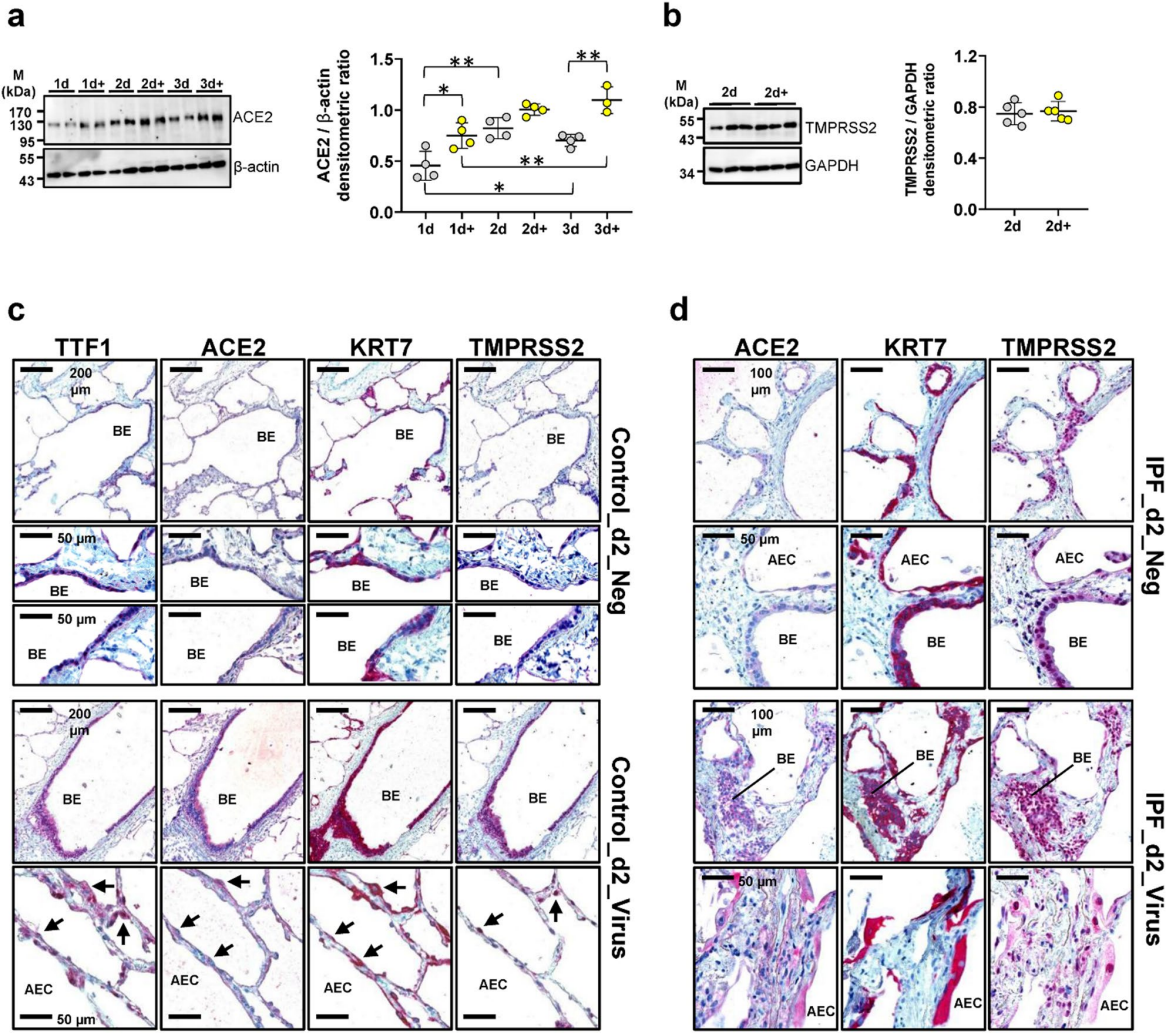
Effect of SARS-CoV-2 infection on expression of viral entry factors ACE2 and TMPRSS2 in human precision-cut lung slices. **a** Human PCLS were treated with vehicle or SARS-CoV-2 (+) for 1d, 2d and 3d, followed by quantitative immunoblot analysis for ACE2. Data are representative for *n* = 2 treatments per timepoint and 2 technical replicates. **b** Quantitative immunoblot-analysis for TMPRSS2 in uninfected (*n* = 5) versus SARS-CoV-2 infected (+) PCLS (*n* = 5) at 2 d.p.i. Data in (**a**) and (**b**) are expressed as means ± SD. ***p < 0.001, **p < 0.01, *p < 0.05 by Bonferroni´s multiple comparisons test. **c** Representative immunohistochemistry for TTF1, ACE2, Cytokeratin-7 (KRT7) and TMPRSS2 in serial sections of uninfected (mock) and SARS-CoV-2-infected PCLS obtained from organ donor lungs at 2 d.p.i. **d** Representative immunohistochemistry for ACE2, KRT7 and TMPRSS2 in serial sections of uninfected (mock) and SARS-CoV-2-infected PCLS obtained from a patient with idiopathic pulmonary fibrosis (IPF) at 2 d.p.i. Abbreviations: BE = bronchial epithelium, AEC = alveolar epithelial cells

Next, we assessed potential effects of LOS and ENA on the mRNA levels of proinflammatory cytokines and chemokines in SARS-CoV-2-infected PCLS. At 1 d.p.i., we observed a robust induction of the “acute phase response cytokine” interleukin-6 (*IL6*) in PCLS cells infected with SARS-CoV-2 compared to uninfected PCLS, which, at 2 d.p.i., was partially reversed in ENA-, but not in LOS-treated PCLS infected with SARS-CoV-2 (Fig. 3a). In contrast, the robust upregulation of the major proinflammatory cytokine *IL1B* by SARS-CoV-2 was significantly diminished by LOS, but not ENA treatment at 2 d.p.i. in infected PCLS (Fig. 3b). Moreover, the SARS-CoV-2-mediated upregulation of the chemokine C-C motif ligand 2 (*CCL2*) could be entirely prevented at 1 d.p.i. in LOS-, but not in ENA-treated PCLS cells infected with SARS-CoV-2 (Fig. 3c), and was completely abolished by both drugs at 2 d.p.i. (Fig. 4c). Furthermore, LOS, but not ENA, resulted in significant reduction of coronavirus-induced expression of C-X-C motif chemokine 2 (*CXCL2*) at 2 d.p.i. in infected PCLS (Fig. 3d). Interestingly, LOS or ENA had no detectable effect on the strong induction of the chemokine *IL8* in SARS-CoV-2-infected cells (Fig. 3e). Expression of tumor necrosis factor-alpha (*TNFA*), which appeared to be slightly, but significantly induced by SARS-CoV-2 at 2 d.p.i., was reduced in response to LOS, but not ENA treatment (Fig. 3f).

A further assessment of the effects of LOS and ENA treatment on these six proinflammatory cytokines in uninfected PCLS (Figures S2a-S2f) revealed that both RAAS-inhibiting drugs did not significantly affect the low basal endogenous expression of *IL6*, *CCL2*, *CXCL2*, *IL8* and *TNFA* in cultured PCLS, whereas *IL1B* appeared to be downregulated in response to LOS treatment (Figure S2b).

**Fig. 5:**
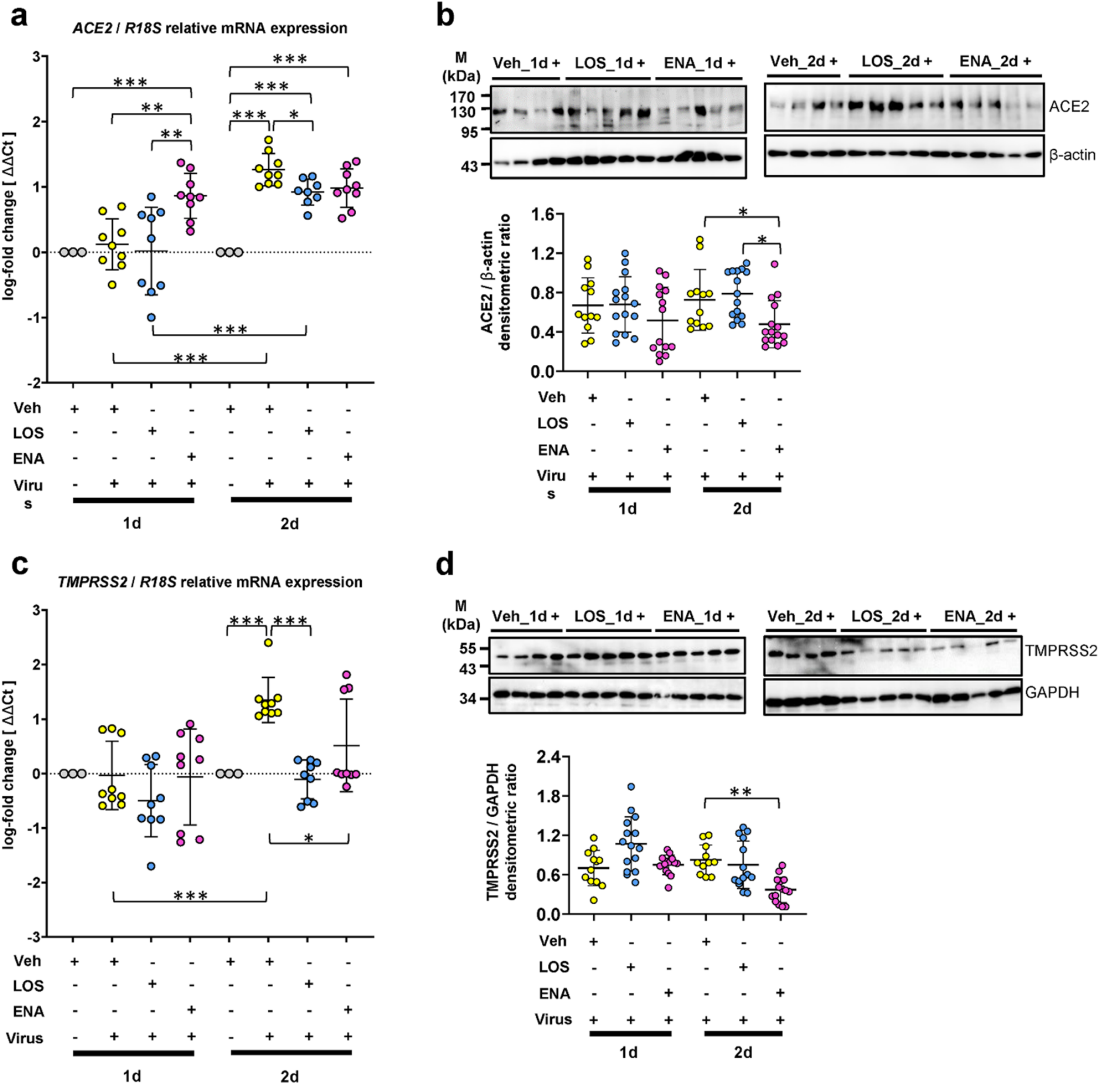
Effects of losartan and enalapril on the expression of SARS-CoV-2 entry factors ACE2 and TMPRSS2 in virus-infected human precision-cut lung slices. Analyzes of gene and protein expression for *ACE2* (**a**, **b**) and *TMPRSS2* (**c**, **d**) in response to SARS-CoV-2 infection at 1 and 2 d.p.i. in the absence (Vehicle) or presence of losartan (LOS) or enalapril treatment (ENA). (**a**) *ACE2* gene expression. (**b**) ACE2 protein expression. (**c**) *TMPRSS2* gene expression. (**d**) TMPRSS2 protein expression. For gene expression analyzes, *R18S* served as reference gene in (**a**) and (**c**). The log_2_ fold-changes are given by the ΔΔCt values, where ΔΔCt = ΔCt[treatment] - ΔCt[untreated (Veh_1d or Veh_2d)]. Data are representative for *n* = 3 distinct treatments, with *n* = 3 technical replicates, and expressed as means ± SD. ***p < 0.001, **p < 0.01, *p < 0.05, by Bonferroni´s multiple comparisons test. Quantitative immunoblot-analyses for ACE2 (**b**) and TMPRSS2 (**d**) are representative for *n* = 3 independent experiments with three different normal human lungs, with *n* = 4–5 distinct treatments per experiment. Data are expressed as means ± SD. **p < 0.01, *p < 0.05, by Dunn´s Multiple Comparisons test

Given the major role of ACE2 and TMPRSS2 in cellular entry of SARS-CoV-2, we next sought to assess potential changes in the respective protein levels in human PCLS cells infected with SARS-CoV-2. ACE2 protein levels were significantly upregulated by SARS-CoV-2 at 1 d.p.i., and further increased until 3 d.p.i., with a modest increase of ACE2 levels being observed also for uninfected PCLS at 2 d.p.i. (Fig. 4a). Protein expression of TMPRSS2 remained unaffected by SARS-CoV-2 at any time (2 d.p.i., Fig. 4b and not shown). Next, we employed IHC to identify the cellular distribution of ACE2 and TMPRSS2 in PCLS cells infected with SARS-CoV-2. Mock-infected PCLS were used as control. In this particular experiment, PCLS from a normal lung (Fig. 4c and Figure S3) and an IPF-lung (Fig. 4d and Figure S4) were used. In addition, serial lung sections were immunostained for KRT7 (marker for simple epithelia) or TTF1 (marker for AECII and bronchial Club cells) to localize the two coronavirus entry-related proteins (Fig. 4c, S8). While uninfected PCLS showed a low cytoplasmic expression of ACE2 in bronchial and alveolar epithelial cells, ACE2 staining was significantly increased in epithelial PCLS cells infected with SARS-CoV-2 at 2 d.p.i. Similar observations were made for TMPRSS2, the expression of which was moderate in uninfected PCLS epithelial cells, but clearly increased upon SARS-CoV-2 infection (Fig. 4c). The SARS-CoV-2-induced upregulation of ACE2 and TMPRSS2 was also observed in epithelial cells of infected IPF-PCLS (Fig. 4d). Next, we assessed potential effects of LOS and ENA on mRNA and protein levels of *ACE2* and *TMPRSS2* in SARS-CoV-2-infected PCLS. Consistent with the protein data shown in Fig. 4, a significant induction of *ACE2* mRNA levels by SARS-CoV-2 was observed at 2 d.p.i. in vehicle- and drug-treated PCLS (compared to uninfected PCLS) (Fig. 5a). Both LOS and ENA were found to slightly reduce the SARS-CoV-2-induced increase of *ACE2* mRNA expression (compared to vehicle-treated cells) (Fig. 5a). Quantitative immunoblot analysis revealed that treatment of SARS-CoV-2-infected PCLS with ENA, but not LOS, significantly reduced ACE2 protein expression at 2 d.p.i. (Fig. 5b). Further, transcript levels for *TMPRSS2* were also upregulated in cultured PCLS infected with SARS-CoV-2 at 2 d.p.i., but SARS-CoV-2-infected PCLS treated with LOS or ENA revealed significantly reduced *TMPRSS2* gene expression at 2 d.p.i. in comparison to untreated infected PCLS (Fig. 5c). At the protein level, however, TMPRSS2 protein expression in coronavirus-infected PCLS was significantly reduced in ENA-treated, but not in LOS-treated cells (Fig. 5d).

**Fig. 6:**
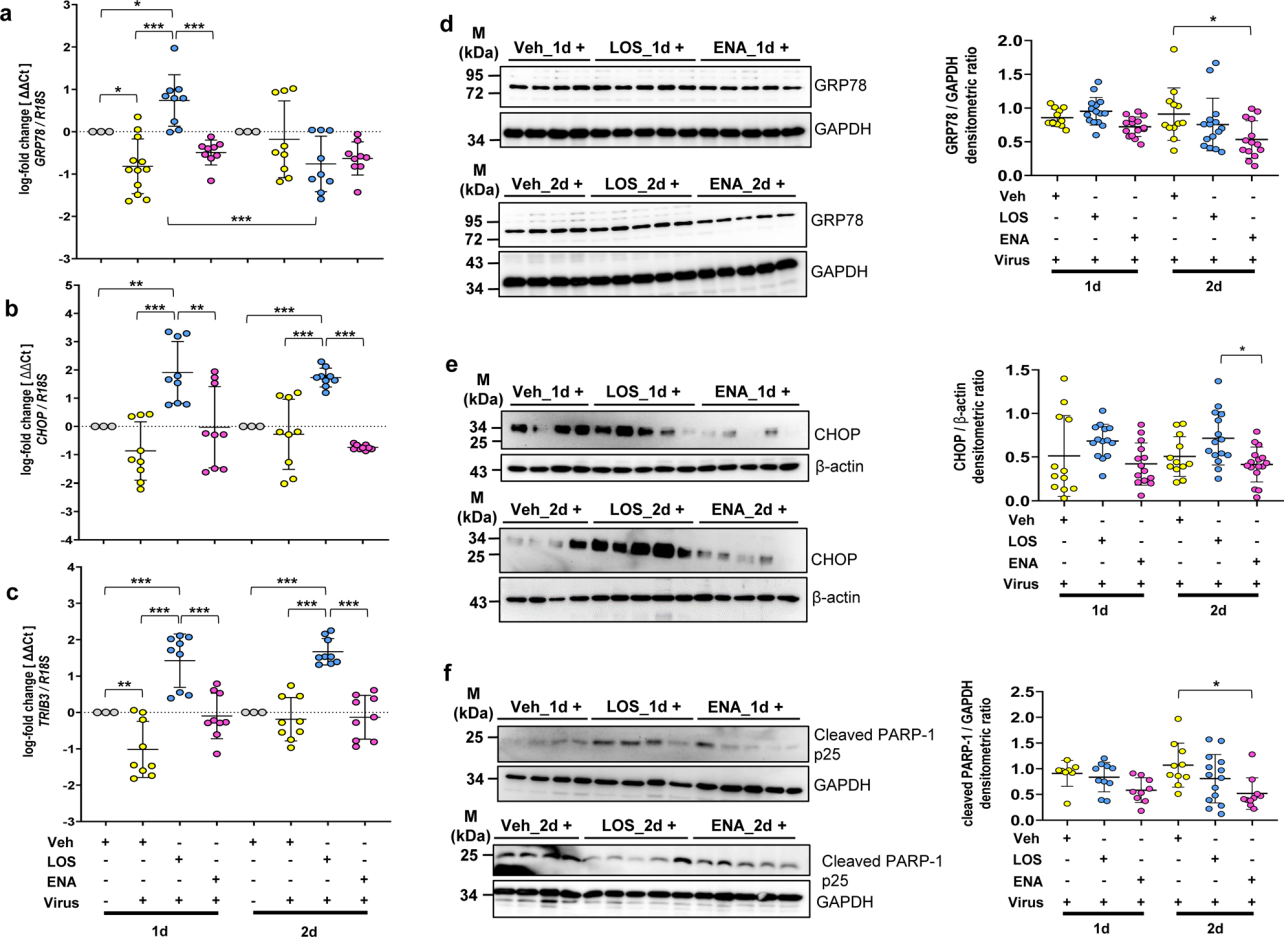
Effects of losartan and enalapril on Endoplasmic Reticulum (ER) stress signaling and apoptosis in SARS-CoV-2-infected human precision-cut lung slices. Analyzes of gene expression for *GRP78* (**a**), *CHOP* (**b**), and *TRIB3* (**c**), in response to SARS-CoV-2 infection at 1 and 2 d.p.i. in the absence (Vehicle) or presence of losartan (LOS) or enalapril treatment (ENA). *R18S* served as reference gene. The log_2_ fold-changes are given by the ΔΔCt values, where ΔΔCt = ΔCt[treatment] - ΔCt[untreated (Veh_1d or Veh_2d)]. Data are representative for *n* = 3 treatments, with *n* = 3 technical replicates. Data are expressed as means ± SD. ***p < 0.001, **p < 0.01, *p < 0.05, by Bonferroni´s multiple comparisons test. Analyzes of protein expression for GRP78 (**d**), CHOP (**e**) and cleaved PARP-1 (**f**) in response to SARS-CoV-2 infection at 1 and 2 d.p.i. in the absence (Vehicle) or presence of losartan (LOS) or enalapril treatment (ENA). Quantitative immunoblot-analyses (**d**) and (**e**) are representative for *n* = 3 independent experiments with three different normal human lungs, with *n* = 4–5 distinct treatments per experiment. (Loading control is similar to Fig. 2d, as gels were run for same samples and probed with several antibodies in a similar manner). In (**f**), data are representative for *n* = 2 independent experiments. All data are expressed as means ± SD. *p < 0.05, by Dunn´s Multiple Comparisons test

Finally, we analyzed the impact of SARS-CoV-2 infection on ER stress signaling and apoptosis, in the absence or presence of LOS and ENA. Much to our surprise, SARS-CoV-2 infection did not result in a significant unfolded protein response (UPR)/ER stress induction in cultured PCLS. Thus, for example, qRT-PCR analyses of typical UPR markers revealed a downregulation of *GRP78*, *CHOP* and tribbles homolog 3 (*TRIB3*) in response to SARS-CoV-2 infection at 1 and 2 d.p.i. in normal cultured PCLS, as compared to uninfected PCLS (Fig. 6a and c). Only the ER chaperone *GRP94* mRNA was slightly upregulated upon SARS-CoV-2 infection (Figure S5). Interestingly, LOS treatment resulted in a significant upregulation of *CHOP* and *TRIB3* mRNA level in SARS-CoV-2-infected PCLS at 1 and 2 d.p.i., whereas ENA did not (Fig. 6b and c).

Further, protein levels of GRP78, GRP94 and CHOP were also not significantly altered upon SARS-CoV-2 infection at 2 d.p.i., as compared to uninfected PCLS. (Figure S6), which was also evident at the mRNA level for these UPR markers after 2d of infection (Fig. 6a and c). Moreover, ENA treatment even significantly reduced the basal GRP78 and CHOP protein levels in SARS-CoV-2-infected PCLS at 2 d.p.i., as compared to vehicle-treated infected PCLS (Fig. 6d and e). In contrast, LOS treatment indicated a trend to upregulate CHOP protein expression vs. vehicle in SARS-CoV-2-infected PCLS at 1 and 2 d.p.i. (Fig. 6e), which was in accordance with the mRNA data (Fig. 6b) and the concomitant induction of the CHOP target gene *TRIB3* at these time points (Fig. 6c).

Finally, in agreement with the protein data obtained for uninfected PCLS (Fig. 1f), ENA treatment reduced the endogenous levels of the apoptosis-indicator cleaved PARP-1 also in SARS-CoV-2-infected PCLS, as compared to vehicle-treated infected PCLS (Fig. 6f). Taken together, our data indicate that ER stress and apoptotic signaling do not appear to play a crucial role in SARS-CoV-2-infected PCLS under the conditions used for such infections in this study.

## Discussion

COVID-19 has emerged as one of the most significant public health challenges of the contemporary era. Despite the development and deployment of several vaccines [22], neutralizing monoclonal antibodies targeting SARS-CoV-2 [23, 24], as well as antiviral and anti-inflammatory therapies [25], the trajectory of the pandemic remains uncertain [26–28]. The continuous evolution of the virus, including the emergence of novel variants such as the recently identified Omicron variant, poses a threat to the efficacy of existing vaccines, which may confer only partial protection against such mutations [29]. Furthermore, certain therapeutic interventions are only effective if administered within a narrow early window following infection, and delays in treatment initiation can contribute to more severe clinical outcomes. Consequently, there is an urgent need to enhance our understanding of how early inhibition of the viral replication cycle—particularly the initial stages involving viral entry into host cells—affects disease progression in humans. Additionally, it is critical to elucidate whether, and to what extent, chronic pharmacological treatments administered to infected individuals influence the clinical course, manifestation, and prognosis of COVID-19.

In this context, particular attention has been directed towards drugs targeting the renin–angiotensin–aldosterone system (RAAS), specifically angiotensin-converting enzyme inhibitors (ACEi) and angiotensin II receptor blockers (ARBs). A key concern under investigation is whether these agents should be discontinued in hypertensive patients with COVID-19 [30, 31], given reports indicating that they may upregulate cardiac expression of ACE2—the cellular entry receptor for SARS-CoV-2 [10, 13, 32]. Such upregulation could potentially facilitate viral entry and dissemination, thereby exacerbating disease severity.

Conversely, SARS-CoV-2 infection has been shown to disrupt the balance between the protective ACE2/Ang-(1–7)/Mas receptor axis and the pro-inflammatory ACE/Ang II/AT1R pathway, favoring the latter and leading to heightened systemic inflammation and pulmonary injury mediated by angiotensin II (Ang II)-dependent signaling [33, 34]. Notably, Ang II has been demonstrated to downregulate ACE2 via AT1R-mediated lysosomal degradation [35] and to induce ACE1 expression through reactive oxygen species (ROS)-dependent activation of ERK/p38 MAPK pathways [36]. These effects may further amplify pro-inflammatory signaling under disease conditions. Taken together, these mechanistic insights provide a rationale for the potential therapeutic benefit of RAAS inhibitors in COVID-19, as they may counteract Ang II-driven pathophysiological processes. However, further clinical and translational research is required to delineate their net impact on patient outcomes and to inform evidence-based treatment guidelines.

There is currently limited knowledge regarding the regulation of pulmonary ACE2 expression in response to RAAS-inhibiting drugs, and no definitive data are available concerning ACE2 modulation under conditions of SARS-CoV-2 infection. Therefore, we investigated the potential therapeutic effects of ENA and losartan LOS using PCLS infected with SARS-CoV-2.

Infection of human PCLS in the absence of RAAS-inhibiting drugs resulted in elevated ACE2 expression, which was quite in contrast to previous studies according to which virus-induced processing and internalization of ACE2 along with the virus [4, 7, 37] and increased proteolytic cleavage of membrane-bound ACE2 by disintegrin and metalloproteinase domain-containing protein 17 (ADAM17) [38] would result in reduced surface ACE2 expression. In agreement with our observations, Ziegler et al. [39] showed that type-I interferons (IFNs) and, to a lesser extent, type-II IFNs increase ACE2 in human (but not mouse) nasal epithelia and lung tissue and suggested that SARS-CoV-2 may exploit the ACE2-mediated tissue-protective response to provide further cellular targets for enhancing virus entry. Moreover, significantly greater numbers of ACE2-positive cells were counted in the lungs from patients who died of COVID-19 or influenza, compared to uninfected controls [40]. Furthermore, Reindl-Schwaighofer et al. [41] observed increased plasma ACE2 activity in severe COVID-19 patients and suggested that this phenomenon may reflect an inflammation-driven, pathophysiological mechanism that aims at counterbalancing the excess of Ang-II. It is important to note that the enzymatic activity and viral receptor property of ACE2 are independent, and the binding of SARS-CoV-2 to ACE2 does not interfere with the enzymatic activity of the latter [42]. Moreover, systemic application of human recombinant soluble ACE2 (*hrs*ACE2) as a decoy receptor for trapping the virus is currently tested as a treatment strategy in severe COVID-19 [43], as inhibition of SARS-CoV-2 infections could be achieved with *hrs*ACE2 in human blood vessel and kidney organoids in vitro [44].

A key and noteworthy finding of our study was that treatment with both LOS and ENA significantly suppressed SARS-CoV-2 replication in human PCLS, with ENA demonstrating a markedly greater antiviral effect compared to LOS. In addition, treatment of uninfected PCLS with either LOS or ENA resulted in a reduction of transmembrane protease TMPRSS2 protein levels. Notably, in SARS-CoV-2-infected PCLS at 2 days post-infection (d.p.i.), ENA treatment led to a pronounced downregulation of both ACE2 and TMPRSS2 protein expression. Importantly, beyond its role as the primary entry receptor, ACE2, the host protease TMPRSS2 plays a critical role in the pathogenesis of SARS-CoV-2. Upon binding of the viral spike (S) protein to ACE2, TMPRSS2 mediates proteolytic cleavage of the spike protein, thereby inducing a conformational change that activates its fusogenic potential. This step is essential for facilitating the fusion of the viral envelope with the host cell plasma membrane, ultimately allowing entry of the viral genome into the cytoplasm [8, 45]. Importantly, our IHC study revealed that TMPRSS2 was always found to co-localize with ACE2 in AEC and the bronchial epithelium of SARS-CoV-2-infected human PCLS. A proof-of-concept study by Iwata-Yoshikawa et al. [46] reported that SARS-CoV failed to replicate in the bronchioles and lungs of *Tmprss2*^(−/−)^ knockout mice. In addition, SARS-CoV-infected *Tmprss2*^(−/−)^ mice indicated reduced expression of inflammatory cytokines as compared to infected wild-type background mice [46]. Thus, the downregulation of ACE2 and TMPRSS2 by ENA may indeed offer a plausible explanation for why this drug was more efficient than LOS in reducing viral replication in human PCLS. However, ACE2-independent mechanisms may play a role in SARS-CoV-2 cellular entry and pathogenesis, because CD147 has been proven as an alternative SARS-CoV-2 receptor enabling virus infection in transgenic human CD147 (*hCD147*) mice [8]. At present, we can only speculate on whether or not LOS has an effect on ACE2/TMPRSS2-independent cellular entry mechanisms. Related to this, it was reported that cathepsin-B/L work independently of TMPRSS2 indicating distinct and mutually exclusive cellular entry routes for SARS-CoV-2 [14].

Notably, SARS-CoV-2 infection was not observed to induce significant apoptosis in ex vivo-cultured PCLS, and ENA-treatment even downregulated the basal protein expression of apoptosis-markers in infected PCLS. Although LOS indeed induced pro apoptotic ER stress marker CHOP in SARS-CoV-2-infected as well as uninfected PCLS, it did not lead to a meaningful induction of apoptosis.

Although this study offers valuable insights into how RAAS-inhibiting drugs may affect SARS-CoV-2 infection in human PCLS, several limitations should be considered. The PCLS model retains key aspects of lung tissue organization, yet it does not fully replicate the complexity of whole-organism responses, including systemic immunity, hormonal signaling, and vascular interactions, which may influence the effectiveness and safety of therapeutic interventions. Additionally, the sample size was limited, potentially restricting the generalizability of the findings across diverse patient populations. The study also focused on short-term responses, leaving the longer-term effects of drug treatment on viral persistence, lung tissue responses, and immune regulation unexplored. While differences were observed between ENA and LOS in modulating host entry factors and cytokine expression, the precise mechanisms behind these observations, especially those unrelated to ACE2-mediated entry, remain unclear. Furthermore, LOS and ENA at concentrations ranging from 100 µM to 1000 µM across various cell types in independent experimental settings and assays were shown to exert more than 80% cell viability, implicating their limited cytotoxicity even at higher concentrations [21]. Although we initially considered to complement the Western blot analyses with cytotoxicity assays, the limited availability of PCLS tissue per donor necessitated a prioritization of mechanistic protein studies over parallel cytotoxicity assessments. These considerations highlight the need for future in vivo studies and clinical investigations to validate and expand upon these fundamental findings.

In conclusion, our findings derived from ex vivo-cultured human PCLS as a model for SARS-CoV-2 infection do not support the hypothesis of detrimental effects associated with ENA or LOS treatment in the context of COVID-19. This interpretation is further corroborated by recently published clinical data from retrospective cohort studies [18, 47] and randomized clinical trials [30, 48], which collectively suggest that the continued use of RAAS-inhibiting medications in hypertensive patients with COVID-19 is not associated with worse clinical outcomes. Notably, our experimental data raise the possibility that ENA and LOS may exert beneficial antiviral effects by attenuating viral replication and spread during the early stages of infection. However, the aforementioned clinical trial was not specifically designed to evaluate this hypothesis, as all enrolled participants were already receiving RAAS inhibitors at the time of infection and were enrolled several days after symptom onset [30]. Therefore, additional preclinical and prospective clinical studies are warranted to validate and expand upon the translational findings presented in our study.

## Supplementary Information

Supplementary Material 1.

## Data Availability

All data generated or analysed during this study are included in the published version of this article and its supplementary information files.
