# Supplementary material for: RAAS antagonists dampen the SARS-CoV-2 infection in ex-vivo cultured human precision-cut lung slices
Source: Respir Res. 2026 Jan 13;27:28. doi: 10.1186/s12931-025-03463-8 (PMC12849188; doi:10.1186/s12931-025-03463-8)
Supplement: Supplementary file 1 — Supplementary Material 1. [file 12931_2025_3463_MOESM1_ESM.zip › Corona MS_SuppInfo_Revison1_Clean.docx]

**RAAS antagonists dampen the SARS-CoV-2 infection in *ex-vivo* cultured human precision-cut lung slices**

Poornima Mahavadi^1,2,^**^#^**, Martina Korfei^1,2,^**^#^**, Christin Müller-Ruttloff^3,4^, Clemens Ruppert^1,2^, Ekaterina Krauss^1,5^, Peter Dorfmüller^2,6^, Stefan Gattenloehner^2,6^, Stefanie Dimmeler^7,8^, Elie El Agha^2,8,9,10^, Saverio Bellusci^2^, Susanne Herold^2,4,8,9,10^, Biruta Witte^2,11^, John Ziebuhr^3,4^, Andreas Guenther^1,2,5,8,12,^**^*^**.

**Online Data Supplement**

**Supplementary Materials and Methods**

**Human lung tissue**

Peripheral lung tissue samples with normal histology were obtained from unaffected lung areas of 5 patients with lung cancer (controls, mean age ± SD: 58.80 ± 8.76 years; 3 females, 2 males) who underwent video-assisted throracic surgery (VATS) during cancer resection. In addition, subpleural normal lung tissue samples were obtained from explanted lungs of two male organ donors aged 58 and 30 years, respectively. Further, peripheral lung tissue of one explanted IPF-lung (male patient, 59 years old) was used for the study. All lung tissues were processed for generation of precision-cut lung slices (PCLS), as described below.

All lung tissue samples were collected and provided by the UGMLC Giessen Biobank (member of the DZL Platform Biobanking). The study protocol was approved by the Ethics Committee of the Justus-Liebig-University Giessen (No. 111/08 and 58/15). IPF diagnoses were always made according to the American Thoracic Society (ATS)/European Respiratory Society (ERS) consensus criteria [1].

**Generation and culture of precision-cut lung slices (PCLS)**

Briefly, peripheral lung tissues were filled with 37°C warm 3% low melting agarose (Sigma, A9414) in DMEM/Ham’s F12 supplemented with 100 U/mL penicillin (Gibco), 100 µg/mL streptomycin (Gibco), and were allowed to cool on ice for 30 minutes for the agarose to solidify. Next, lung tissues were cut with a Microm HM 650 V vibratome (Thermo Scientific) to a thickness of 300 μm. The PCLS were then cultured in 6-well-plates (35 mm diameter per well) containing RPMI medium without phenol red supplemented with 2% fetal bovine serum (FBS) (Sigma, F0804), ITS-solution (5 µg/mL insulin, 10 µg/mL transferrin, 30 nM sodium selenite; PAN Biotech, P07-03100), 100 U/mL penicillin, 100 µg/mL streptomycin, 2 mM L-glutamine (Gibco) and 2.5 μg/mL amphotericin B (Sigma, A9528), at 37°C in a 5% CO_2_ atmosphere, with one slice per well. The medium was changed every day.

**Viral infection**

SARS-CoV-2 (NCBI SRA accession number SRX9907172 and SRX8975039) was propagated and titrated by plaque assay on Vero E6 cells, to determine plaque forming units/ml (pfu/ml). Human PCLS were infected with 1 × 10^5^ pfu per PCLS at 33°C. Mock-infected tissue – i.e. tissue samples incubated with medium without virus served as negative controls. PCLS were harvested at 1, 2, and 3 days post infection (d.p.i.), for mRNA-, protein- and immunohistochemical (IHC) analyses. For protein analyses by immunoblotting, PCLS were washed with PBS and transferred to 250 µL lysis buffer per PCLS (see full details in the chapter western blot analysis), followed by homogenization using the MagNa Lyser (Roche) and Precelleys Tissue Homogenizer. Lung homogenates had to be subjected to thermal inactivation of SARS-CoV-2 at 70°C for 10 min. For RNA isolation, PCLS were transferred each to 1 mL TRIzol™ reagent (Invitrogen, #15596026) containing phenol and guanidinium thiocyanate. For IHC analyses, PCLS were transferred to 4% (w/v) phosphate-buffered paraformaldehyde for fixation (3-4h). Thermal inactivation of the virus was not necessary for RNA isolation or prospected IHC analyses.

**Drug treatments in the absence or presence of viral infection**

Cultured human PCLS were treated for 6d with 0.33% (v/v) DMSO (vehicle), 300 µM losartan (Sigma, #61188) or 300 µM enalaprilat dihydrate (Selleckchem, S1657), with daily change of the culture medium, followed by harvest of PCLS for mRNA- (n=3), IHC- (n=3) and protein analyses (n=4-5).

To test the effects of RAAS-inhibiting drugs on SARS-CoV-2 infection, PCLS were pre-treated for 5d with vehicle, 300 μM losartan or 300 µM enalapril prior to infection, followed by infection of PCLS with vehicle/mock or SARS-CoV-2 at 1 × 10^5^ pfu/PCLS at 33°C on the 6^th^ day and incubation for 1d or 2d in the "further" presence or absence of losartan and enalapril. Thereafter, PCLS from each condition were harvested for mRNA-, IHC- and protein analyses with the same n-numbers as described above. This main experiment was performed four times with PCLS from unaffected lung tissue of 4 individuals. Figure 1 (main manuscript) indicates the flowchart of the main experiments.

**RNA isolation, Reverse Transcription, and quantitative Real-Time Polymerase Chain Reaction (qRT-PCR, qPCR)**

Total RNA was isolated from PCLS by phenol-chloroform extraction using the TRIzol™ reagent and Precelleys Tissue Homogenizer (Peqlab, Germany) for lysis, according to the protocol of the manufacturer. The total RNA was precipitated with isopropanol and washed with 75% ethanol, and was dried at air. RNA pellets were dissolved in 20-50 µL endotoxin-free H_2_O. The purity and quantity of the isolated RNA was determined by spectrophotometry at 260/280 nm using NanoDrop 2000c photometer (PeqLab).

Complementary DNA (cDNA, 1 µg) was synthesized by reverse transcription (RT) using 1 µg total RNA, with use of the Omniscript-RT-Kit (Qiagen, #205113) and 10 µM random hexamers (Invitrogen, #N8080127) per reaction in a volume of 20 µL. Quantitative (q)RT-PCR reactions were set-up in a 96-well format containing iTaq Universal SYBR^®^ Green Supermix (Bio-Rad Laboratories, Germany, #1725124), 500 nM of each forward and reverse primer (Metabion International AG, Germany), and 5-10 ng of cDNA sample and H_2_O in a 10-μL reaction volume. Real-time fluorescence of PCR products was detected using a Mx3000P qPCR System (Agilent Technologies, CA, USA) using the following thermocycling conditions: 1 cycle of 95°C for 2 min (“Hot-Start”) in the first segment; followed by "3-step-cycling" with 40 cycles of 95°C for 15 s (denaturation), and 60°C for 20 s (primer annealing), and 72°C for 1 min (extension) in the second segment; and finally 1 cycle of 95 °C for 15 s and 60 °C for 1 min followed by 0.5 °C increments at 2 s/step back to 95 °C for 15 s in the third segment (melting curve). The complete list of human gene-specific primers used is given in Supplementary Table S2. The list of SARS-CoV-2-specific primers is given in Supplementary Table S3. Each gene was normalized to the expression of the gene encoding 18S ribosomal RNA (*R18S*). Each qPCR was assessed in triplicates. From real-time PCR data, ΔCt values were calculated as ΔCt = Ct[reference gene]-Ct[target gene]. The relative changes in transcript level upon treatments are given by the log_2_ fold-changes, known as ΔΔCt values, where ΔΔCt = ΔCt[treatment] - ΔCt[untreated].

The specificity of all primer pairs was also evaluated by using qualitative RT-PCR followed by agarose gel-electrophoresis and detection through UV imager (Gel-Doc XR^+^ system, Bio-Rad Laboratories GmbH, Munich, Germany). All primer pairs led to the amplification of a single fragment with the appropriate size.

Supplementary Figure E7 shows a qualitative RT-PCR analysis for detection of SARS-CoV-2 infection (by using three distinct primer pairs for SARS-CoV-2 *N* gene) in the infected human PCLS.

**Western Blot analysis**

For preparation of lung homogenates, cultured PCLS were washed with PBS and transferred per slice to 250 µL lysis buffer containing 50 mM Tris-HCl (pH 7.5), 150 mM NaCl, 1% (w/v) Triton X-100, 0.5% (w/v) Na-deoxycholate, 5 mM EDTA, and 1× Halt™ Protease and Phosphatase Inhibitor Cocktail (ThermoFisher Scientific, #78440), followed by homogenization using the MagnaLyser and Precelleys Tissue Homogenizer, and incubated on ice for 2 h. Cell debris was removed from crude extracts by centrifugation at 13000 rpm and 4°C for 10 min. The resulting lung homogenates were frozen at -80°C until used.

Protein concentrations were determined with the Pierce BCA protein assay kit (ThermoFisher Scientific). Lung homogenates were then diluted (1:3) in 4× SDS sample buffer [final concentration: 2% (w/v) SDS; 2.5% (v/v) β-mercaptoethanol; 10% (v/v) glycerol; 12.5 mM Tris-HCl (pH 6.8), and 0.1% (w/v) bromophenol blue] and denatured by heating at 99°C for 15 min.

Protein extracts (10-15 μg per lane) were subjected for separation to 8-15% SDS-PAGEs, followed by transfer onto PVDF membranes (Millipore) in a semi-dry blotting chamber according to the manufacturer ́s protocol (Bio-Rad). Obtained immunoblots were then blocked by incubating at room temperature (RT) for 1h in blocking buffer [1× tris-buffered saline (TBS; 50 mM tris-HCl with pH 7.5, 50 mM NaCl) containing 5% (w/v) nonfat dried milk and 0.1% (v/v) tween 20], followed by immunostaining for the proteins of interest. Blots were incubated with the respective primary antibodies (diluted in blocking buffer) overnight at 4°C with gentle shaking. In the following, the primary antibodies used for immunoblotting are listed, including the sources and dilutions: goat polyclonal for human ACE2 (1:300, R&D systems, AF933), rabbit monoclonal for human TMPRSS2 (1:500, Abcam, ab92323), rabbit monoclonal for human CHOP (1:300, CST, #5554), rabbit polyclonal for mouse GRP78 (1:500, Abcam, ab21685), rat monoclonal for chicken GRP94 (1:300, Santa Cruz Biotech., sc-32249), rabbit monoclonal for cleaved human PARP-1 (1:500, Abcam, ab32064), and rabbit polyclonal to SARS-CoV-2 Nucleocapsid (N) protein (1:300, Rockland, #200-401-A100). The blots were then washed four times in 1× TBS containing 0.1 % (w/v) tween 20, and incubated with respective horseradish peroxidase (HRP)-conjugated secondary antibodies [rabbit anti-mouse-IgG, rabbit anti-goat-IgG, or swine anti-rabbit IgG, all from DakoCytomation, Germany; rabbit anti-rat IgG from Abcam (ab6734)], all diluted 1:1000 in blocking buffer, for 2 hours at RT. After four washes, blot membranes were developed with the Immobilon Western Chemiluminescent HRP substrate (Millipore), and emitted signals were detected with a chemiluminescence imager (Intas ChemoStar, Intas, Germany).

Thereafter, blots were re-probed with antibodies against the loading control GAPDH (mouse monoclonal for human GAPDH, Santa Cruz Biotech., sc-47724, diluted 1:1000) or β-actin (mouse monoclonal for human β-actin, Abcam, ab8226, diluted 1:2000). For quantification, band intensities were quantified by densitometry using ImageJ software (Version 1.53a, NIH). The band densities were normalized to loading controls.

Due to small amount of whole protein isolated from the small PCLS, some immunoblots were probed for two research targets [e.g. SARS-CoV-2 (N) protein (46 kDa) together with GRP78 (78 kDa) on the same blot], followed by re-probing of blots with loading controls.

**Immunohistochemistry (IHC)**

Cultured human PCLS were placed in 4% (w/v) paraformaldehyde for fixation for 3-4h and thereafter washed in PBS, followed by dehydration and paraffin embedding. Sections (3 µm) were cut and mounted on positively charged glass slides. Paraffin-embedded lung tissue sections were then deparaffinized in xylene and rehydrated in graded alcohol. Antigens were retrieved by cooking the sections for 5 min in 10 mM/L citrate buffer (pH 6.0) using microwave irradiation (800 W). Thereafter, sections cooled down for 20 min at RT, followed by repeated cooking (800 W, 5 min) and cooling (20 min at RT). This procedure was performed three times. ZytoChem-Plus AP-Permanent Red-kit (Zytomed Systems, Germany, #AP008RED) was used for immunohistochemical localization of ACE2, TMPRSS2, SARS-CoV-2, Cytokeratin-7 (KRT7) and TTF1 in formalin-fixed, paraffin-embedded lung tissue sections, according to the manufacturer´s instructions. In the following, the primary antibodies used for IHC are listed, including the sources and dilutions: rabbit monoclonal for human KRT7 (1:400, Abcam, ab68459), rabbit monoclonal for human TTF1 (1:100, Abcam, ab76013), mouse monoclonal for human ACE2 (1:50, R&D systems, MAB933), rabbit monoclonal for human TMPRSS2 (1:500, Abcam, ab92323), and rabbit monoclonal to SARS-CoV-2 (2019-nCoV) Spike S1 protein (1:50, Sino Biological, #40150-R007).

In general, sections were incubated overnight at 4°C with primary antibodies, which were diluted in PBS containing 2% (w/v) BSA. Control sections were treated with PBS-BSA alone to determine the specificity of the staining. Detection was performed with a polyvalent secondary biotinylated antibody (rabbit, mouse, rat, guinea pig, provided by the ZytoChem Plus AP Kit, 20 min incubation) followed by incubation with AP-conjugated streptavidin (20 min). Sections were then developed with Permanent Red substrate solution, and the reaction was terminated by washing in distilled water. The stained sections were counterstained with hemalaun and mounted in Glycergel (DakoCytomation, #C0563). Lung tissue sections were scanned with a scanning device (Nano-Zoomer, Hamamatsu), and examined histopathologically using the ´NDP.view2 software´ at 100×, 200×, and 400× original magnification.

**Supplementary Table S1. Data of patients who underwent video-assisted thoracic surgery (VATS) for lung cancer resection. Unaffected areas from the following patients were obtained for this study**.

| **Patient** | **Age** | **Gender** | **Medications 12 hours before VATS** |
| --- | --- | --- | --- |
| VATS#1 | 47 | f | Levothyroxine, Ramipril |
| VATS#2 | 56 | f | Tilidin, Pantoprazole, Ibuprofen |
| VATS#3 | 58 | f | n/a |
| VATS#4 | 71 | m | Esomeprazole, Acetylsalicylic acid, Carvedilol, Candesartan, Simvastatine |
| VATS#5 | 62 | m | Alendronic acid |

Supplementary Table S2: Primers used in quantitative RT-PCR (homo sapiens)

| **Gene (human)** | **Forward primer** | **Reverse primer** |
| --- | --- | --- |
| *R18S* | 5´-GACTCAACACGGGAAACCTC-3´ | 5´-ATGCCAGAGTCTCGTTCGTT-3´ |
| *ACE2* | 5´-AAACATACTGTGACCCCGCAT-3´ | 5´-CCAAGCCTCAGCATATTGAACA-3´ |
| *TMPRSS2* | 5´-CTGCCAAGGTGCTTCTCATT-3´ | 5´-CTGTCACCCTGGCAAGAATC-3´ |
| *IL6* | 5´-AACCTGAACCTTCCAAAGATGG-3´ | 5´-ATCTGGACAGCTCTGGCTTG-3´ |
| *IL1B* | 5´-ATCACTGAACTGCACGCTCC-3´ | 5´-TTGTTCTCCATATCCTGTCCC-3´ |
| *IL8* | 5´-GGCTCTCTTGGCAGCCTTC-3´ | 5´-GGTTTGGAGTATGTCTTTATGCAC-3´ |
| *TNFA* | 5´-GCCTCTTCTCCTTCCTGATCG-3´ | 5´-AGCTTGAGGGTTTGCTACAACA-3´ |
| *CCL2* | 5´-TTCCCCTAGCTTTCCCCAGA-3´ | 5´-TCCCAGGGGTAGAACTGTGG-3´ |
| *CXCL2* | 5´-CTGCGCCCAAACCGAAGTCATA-3´ | 5´-TTCAGGAACAGCCACCAATAAGC-3´ |
| *GRP78* | 5´-TGGAATGACCCGTCTGTGC-3´ | 5´-CTTTGGTTGCTTGGCGTTGG-3´ |
| *GRP94* | 5´-GTCTCAGCGCCTGACAGAAT-3´ | 5´-GTCTCTGATCAGCGGGTGTC-3´ |
| *TRIB3* | 5´-CTGGTACCCAGCTCCTCTAC-3´ | 5´-GAATCATCTGGCCCAGTCAG-3´ |
| *CHOP* | 5´-ACTCTCCAGATTCCAGTCAGAG-3´ | 5´-GCCTCTACTTCCCTGGTCAG-3´ |
| *CIP1* | 5´-GATGGAACTTCGACTTTGTCAC-3´ | 5´-GGCACAAGGGTACAAGACAG-3´ |

Supplementary Table S3: Primers used in qualitative/quantitative RT-PCR (SARS-CoV-2 diagnostics)

| **SARS-CoV-2 (*N* gene)** | **Forward primer** | **Reverse primer** |
| --- | --- | --- |
| *N_Sarbeco* [2] | 5´-GACTCAACACGGGAAACCTC-3´ | 5´-ATGCCAGAGTCTCGTTCGTT-3´ |
| *2019-nCoV_N1* [3] | 5´-GACCCCAAAATCAGCGAAAT-3´ | 5´-TCTGGTTACTGCCAGTTGAATCTG-3´ |
| *2019-nCoV_N2* [3] | 5´-TTACAAACATTGGCCGCAAA-3´ | 5´-GCGCGACATTCCGAAGAA-3´ |

**Supplementary References**

1. Raghu G, Collard HR, Egan JJ, Martinez FJ, Behr J, Brown KK, Colby TV, Cordier JF, Flaherty KR, Lasky JA, Lynch DA, Ryu JH, Swigris JJ, Wells AU, Ancochea J, Bouros D, Carvalho C, Costabel U, Ebina M, Hansell DM, Johkoh T, Kim DS, King TE, Jr., Kondoh Y, Myers J, Muller NL, Nicholson AG, Richeldi L, Selman M, Dudden RF, Griss BS, Protzko SL, Schunemann HJ. An official ATS/ERS/JRS/ALAT statement: Idiopathic pulmonary fibrosis: Evidence-based guidelines for diagnosis and management. *Am J Respir Crit Care Med* 2011;183:788-824.

2. Corman VM, Landt O, Kaiser M, Molenkamp R, Meijer A, Chu DK, Bleicker T, Brunink S, Schneider J, Schmidt ML, Mulders DG, Haagmans BL, van der Veer B, van den Brink S, Wijsman L, Goderski G, Romette JL, Ellis J, Zambon M, Peiris M, Goossens H, Reusken C, Koopmans MP, Drosten C. Detection of 2019 novel coronavirus (2019-nCoV) by real-time RT-PCR. *Euro Surveill* 2020;25(3):2000045.

3. Ref: SARS-CoV-2 N1+N2 Assay Kit (#222015, Qiagen).

**Supplementary figure legends**

**Figure S1. Gene expression of SARS-CoV-2 entry factors, ER stress markers and cell cyclus-arrest marker *CIP1* in cultured precision-cut lung slices (PCLS) in response to treatment with losartan or enalapril.** Cultured PCLS were incubated for 6d with vehicle [Veh, 0,33% (v/v) DMSO], losartan (LOS, 300 µM) or enalapril (ENA, 300 µM). PCLS were harvested for RNA isolation, and analyzed by qRT-PCR for *ACE2* (a), *TMPRSS2* (b), *GRP78* (c), *GRP94* (d), *CHOP* (e), *TRIB3* (f) and *CIP1* (g). From real-time PCR data, ΔCt values were calculated as ΔCt = Ct[reference]-Ct[target gene] using the Ct values of *R18S* (reference gene). The log_2_ fold-changes are given by the ΔΔCt values, where ΔΔCt = ΔCt[treatment] - ΔCt[untreated (Veh)]. Data are representative for n=3 distinct treatments, with n=3 technical replicates, and are expressed as means ± SD. ***p<0.001, **p<0.01, *p<0.05, by Bonferroni´s multiple comparisons test.

**Figure S2. Gene expression of proinflammatory cytokines in cultured precision-cut lung slices (PCLS) in response to treatment with losartan or enalapril.** Cultured PCLS were incubated for 6d with vehicle [Veh, 0,33% (v/v) DMSO], losartan (LOS, 300 µM) or enalapril (ENA, 300 µM). PCLS were harvested for RNA isolation, and analyzed by qRT-PCR for *IL6* (a), *IL1B* (b), *CCL2* (c), *CXCL2* (d), *IL8* (e), and *TNFA* (f). From real-time PCR data, ΔCt values were calculated as ΔCt = Ct[reference]-Ct[target gene] using the Ct values of *R18S* (reference gene). The log_2_ fold-changes are given by the ΔΔCt values, where ΔΔCt = ΔCt[treatment] - ΔCt[untreated (Veh)]. Data are representative for n=3 distinct treatments, with n=3 technical replicates, and are expressed as means ± SD. ***p<0.001, *p<0.05, by Bonferroni´s multiple comparisons test.

**Figure S3. Expression and localization of viral entry factors ACE2 and TMPRSS2 in uninfected and SARS-CoV-2 infected human PCLS generated from organ donors.** Representative immunohistochemistry for TTF1, ACE2, Cytokeratin-7 (KRT7) and TMPRSS2 in serial sections of uninfected (mock) and SARS-CoV-2-infected PCLS obtained from organ donor lungs at 2 d.p.i.. Abbreviations: BE = bronchial epithelium, AEC = alveolar epithelial cells.

**Figure S4. Expression and localization of viral entry factors ACE2 and TMPRSS2 in uninfected and SARS-CoV-2 infected human PCLS generated from an idiopathic pulmonary fibrosis (IPF)-lung.** Representative immunohistochemistry for ACE2, KRT7 and TMPRSS2 in serial sections of uninfected (mock) and SARS-CoV-2-infected PCLS obtained from an IPF-lung at 2 d.p.i.. Abbreviations: BE = bronchial epithelium, AEC = alveolar epithelial cells.

**Figure S5. Effects of losartan and enalapril on *GRP94* gene expression in SARS-CoV-2-infected human precision-cut lung slices.** Cultured human PCLS were pretreated for 5d with vehicle [Veh, 0,33% (v/v) DMSO], losartan (LOS, 300 µM) or enalapril (ENA, 300 µM), followed by infection of PCLS with vehicle or SARS-CoV-2 for 1d or 2d in the presence or absence of losartan and enalapril. PCLS were harvested for RNA isolation, and analyzed by qRT-PCR for *GRP94*. From real-time PCR data, ΔCt values were calculated as ΔCt = Ct[reference]-Ct[target gene] using the Ct values of *R18S* (reference gene). The log_2_ fold-changes are given by the ΔΔCt values, where ΔΔCt = ΔCt[treatment] - ΔCt[untreated (Veh_1d or Veh_2d)]. Data are representative for n=3 distinct treatments, with n=3 technical replicates, and are expressed as means ± SD. ***p<0.001, *p<0.05, by Bonferroni´s multiple comparisons test.

**Figure S6. Effect of SARS-CoV-2 infection on protein expression of ER stress markers.** Comparative immunoblot analysis for GRP78 and GRP94 (A, B), and for CHOP (C) in uninfected (n=6) versus SARS-CoV-2 infected (+) PCLS (n=6) at 2 d.p.i.. Data are expressed as means ± SD. Results did not reach statistical significance. (D) Human PCLS were treated with vehicle or SARS-CoV-2 (+) fir 1d, 2d and 3d, followed by quantitative immunoblot analysis for GRP94. Data are expressed as means ± SD, and representative for n = 2 treatments per timepoint.

**Figure S7. Qualitative RT-PCR for detection of SARS-CoV-2-infection in infected human PCLS.** Cultured human PCLS were pretreated for 5 days with vehicle [Veh, 0,33% (v/v) DMSO], losartan (LOS, 300 μM) or enalapril (ENA, 300 μM), followed by infection of PCLS with vehicle or SARS-CoV-2 for 1d or 2d in the further presence or absence of losartan and enalapril. PCLS were harvested for RNA isolation, and analysed by qualitative reverse transcription-polymerase chain reaction (RT-PCR) for SARS-CoV-2 infection using specific primers from Corman et al, 2020 (N_Sarbeco [E2]) or published by Qiagen [E3]. Each PCR reaction was performed with 100 ng reverse-transcribed complementary DNA, 1 μM of each forward and reverse primer, 200 μM of each dNTP (#R0192, Thermo Scientific) and 0.4 μL Phire-Hot-Start-II-DNA-Polymerase (#F-122L, Thermo Scientific) in a volume of 20 μL. R18S was used as reference gene. As control experiment, PCR reactions of RNA samples without reverse transcriptase (-RT) were performed. Equal aliquots (8 μL) of the PCR products were electrophoresed through a 2% (w/v) agarose gel containing ethidium bromide in 1× tris-acetate-EDTA (TAE) buffer. To provide additional virus-negative controls, RNA was isolated from plasmid-transfected epithelial MLE12 and A549 cells, as well as from explanted lungs of organ donors or patients with idiopathic pulmonary fibrosis (IPF), non-specific interstitial pneumonia (NSIP) and chronic obstructive pulmonary disease (COPD) (explanted in 2003-2005). Complementary DNA (cDNA, 1 μg) was synthesized for all samples shown here at the same time by reverse transcription (RT) using 1 μg total RNA, with use of Random Hexamers and the Omniscript-RT-Kit (Qiagen).

**Figure S8. Representative immunohistochemistry for cytokeratin-7 (KRT7, marker for simple epithelia) in lung tissue sections of two patients who died from COVID-19.** The lung of patient 1 revealed KRT7-expressing alveolar epithelial cells (AECs, red staining), whereas the lung of patient 2 revealed total loss of AECs and dense fibrotic remodeling.
