# Supplementary figures and images for: RAAS antagonists dampen the SARS-CoV-2 infection in ex-vivo cultured human precision-cut lung slices
Source: Respir Res. 2026 Jan 13;27:28. doi: 10.1186/s12931-025-03463-8 (PMC12849188; doi:10.1186/s12931-025-03463-8)

Figure 1

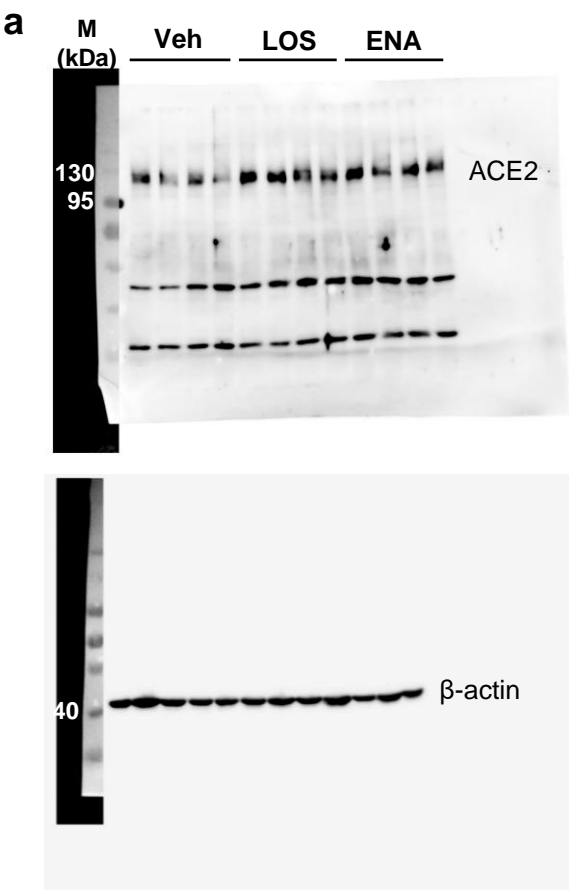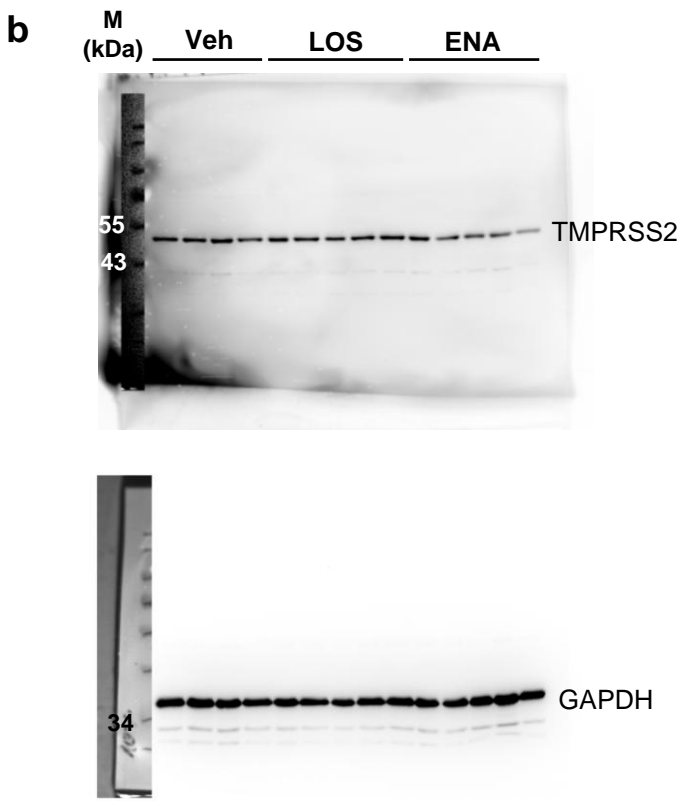

Figure 1

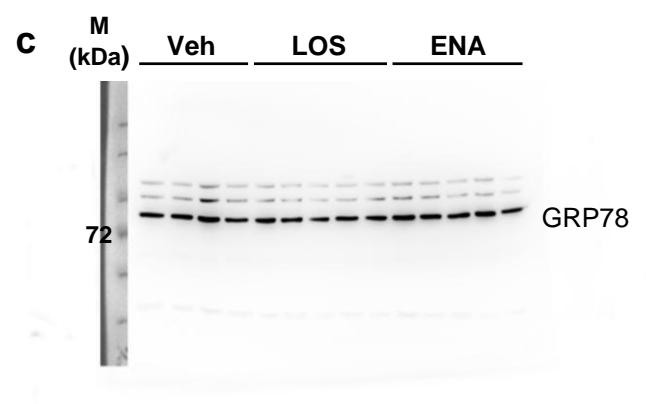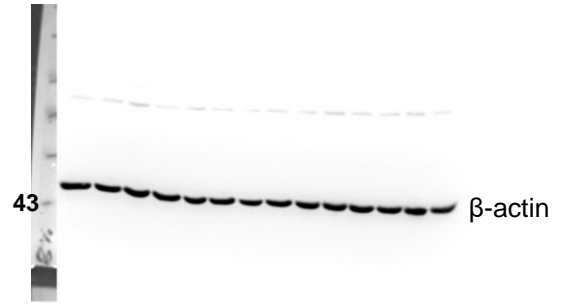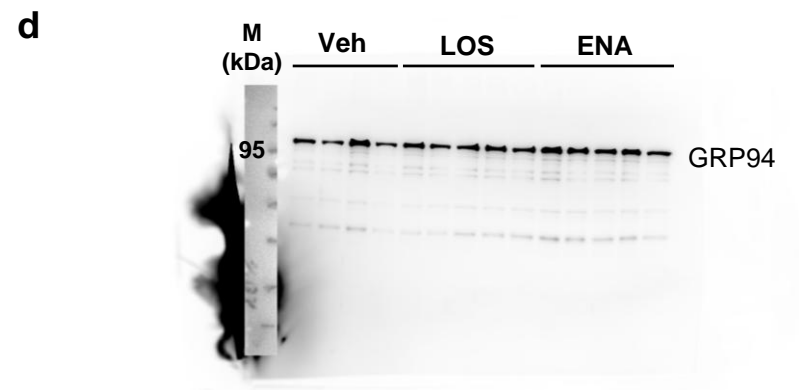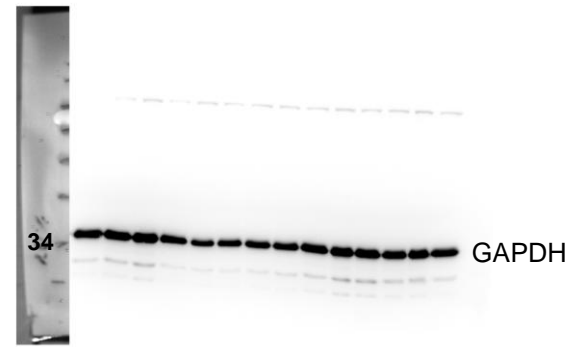

**Figure 1**

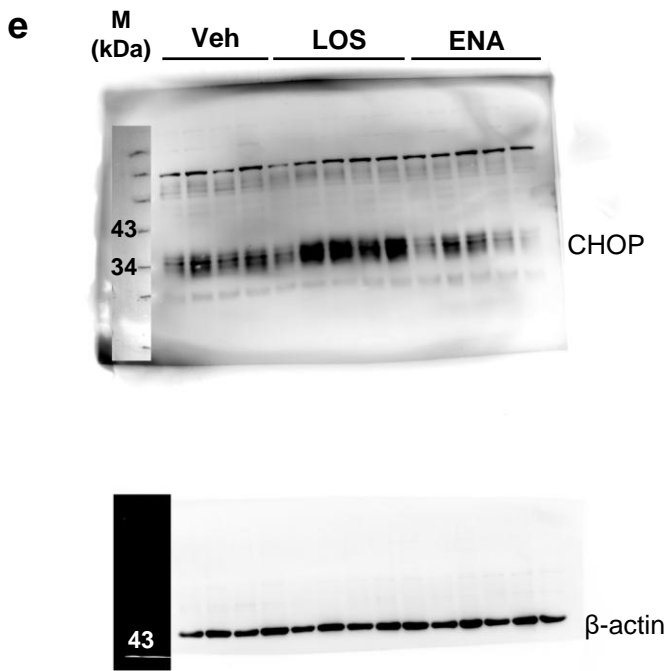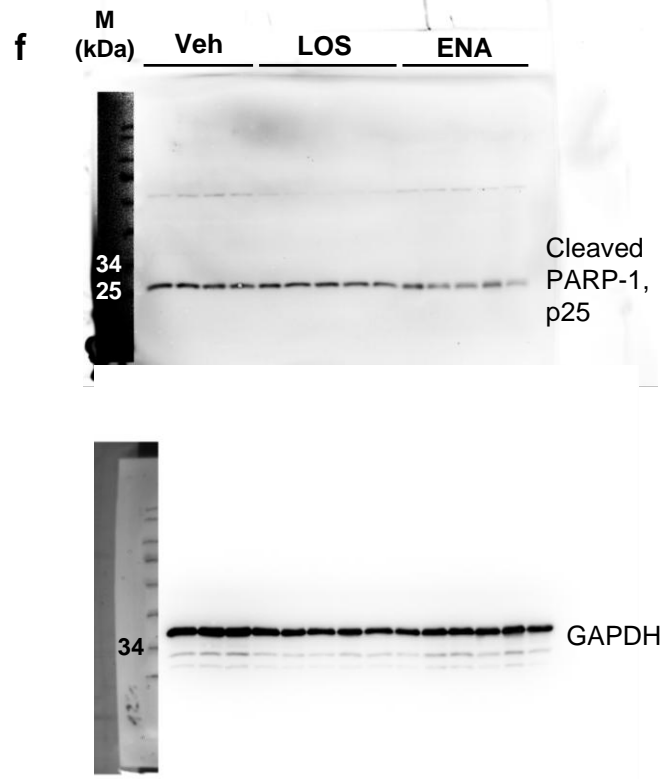

Figure 2

c

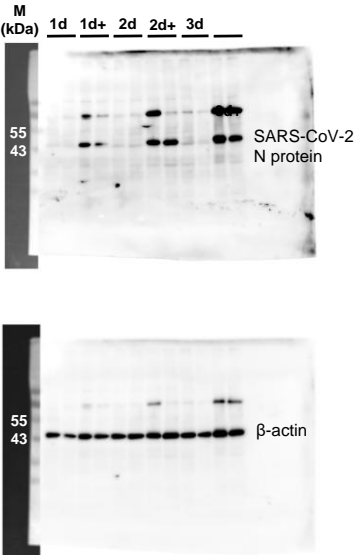

d

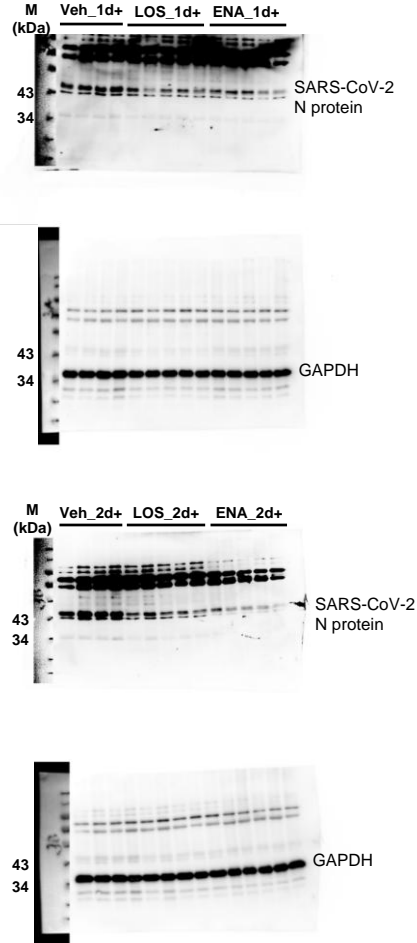

Figure 4

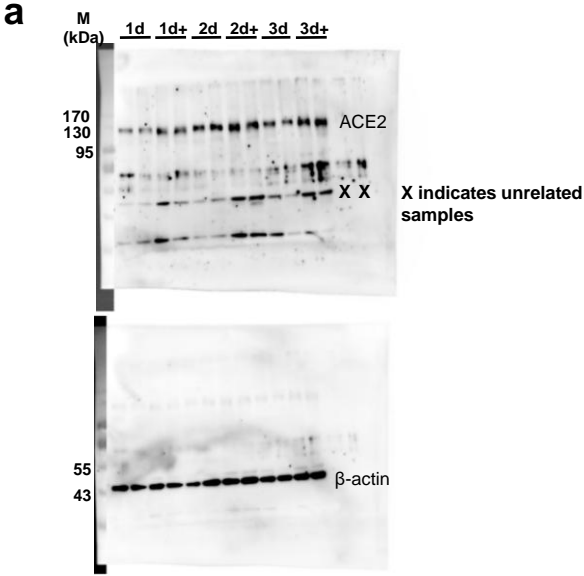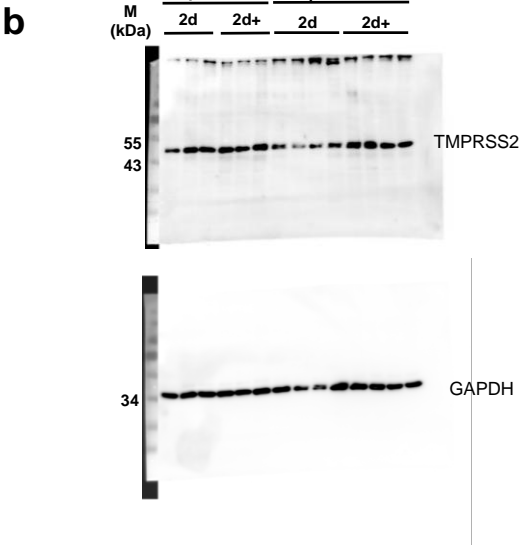

Figure 5

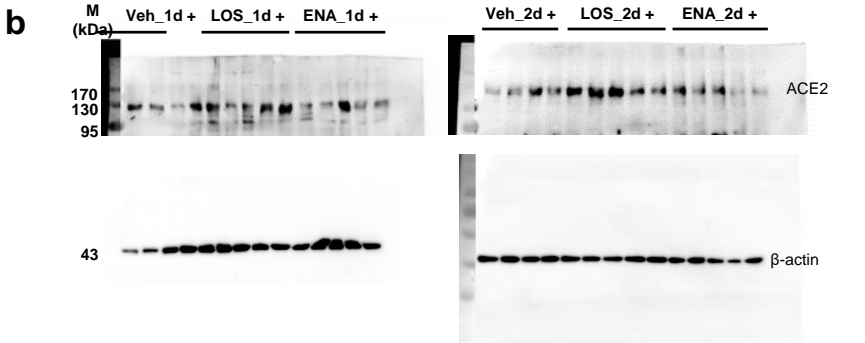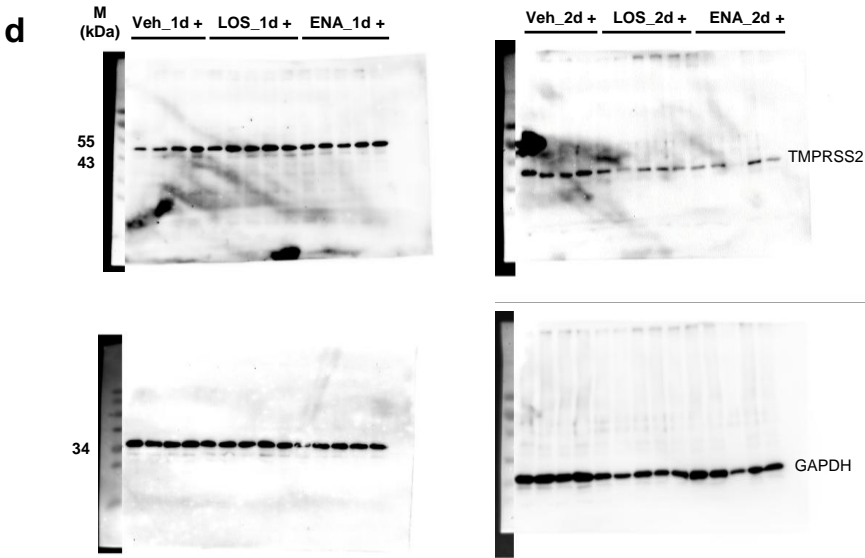

Figure 6

d

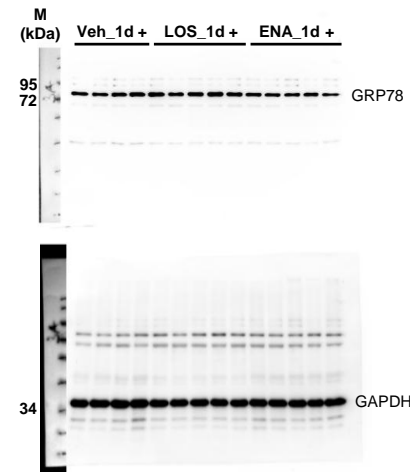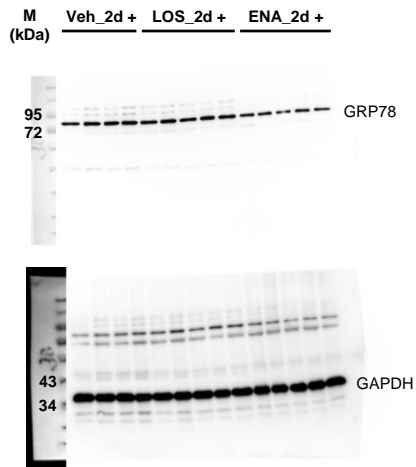

e

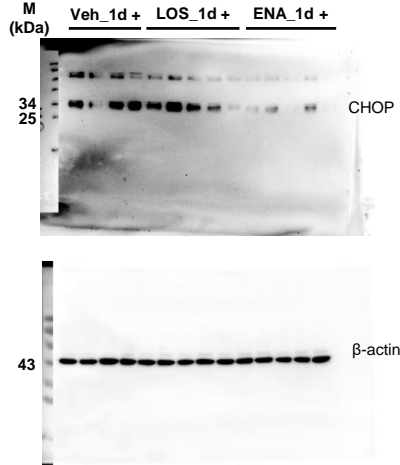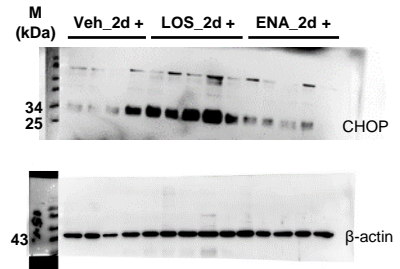

f

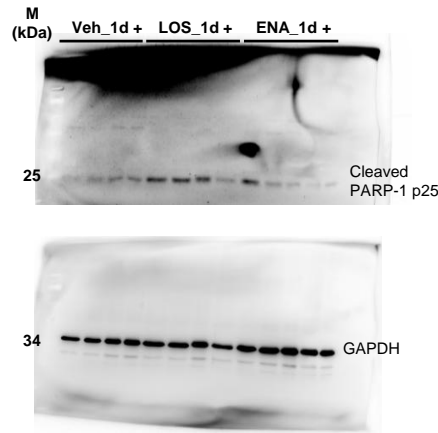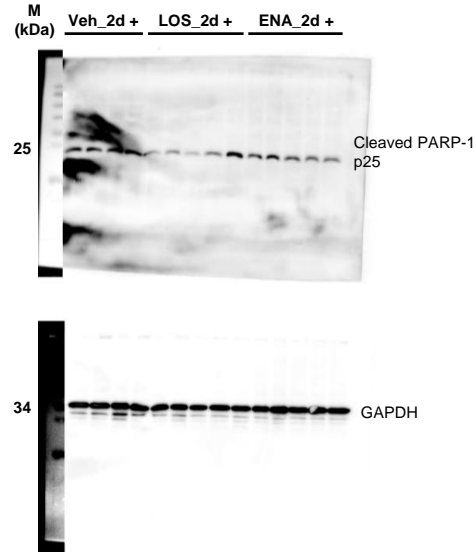

**Figure S6**

**b**

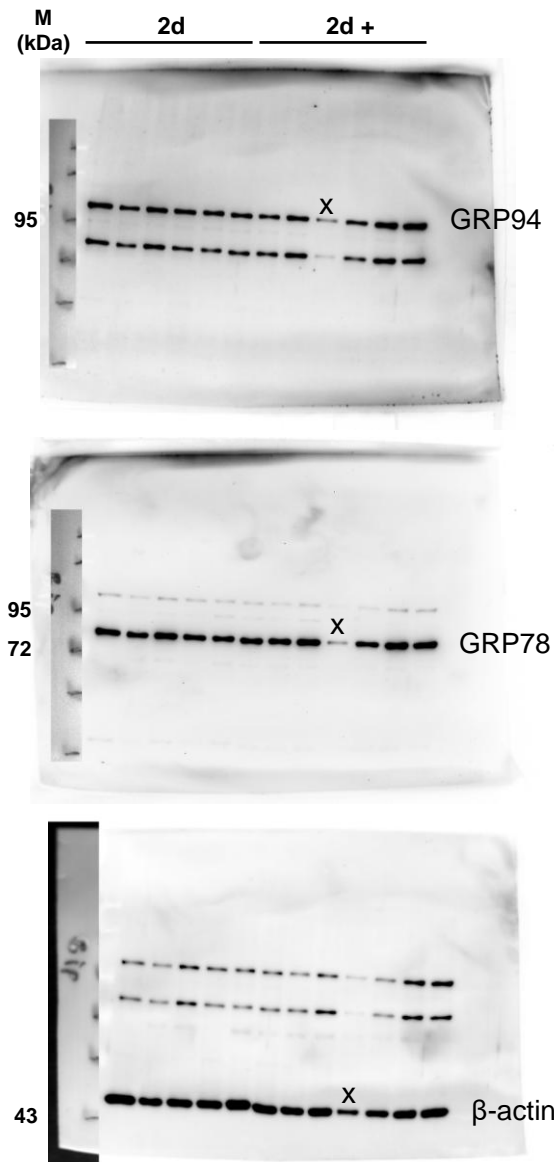

**c**

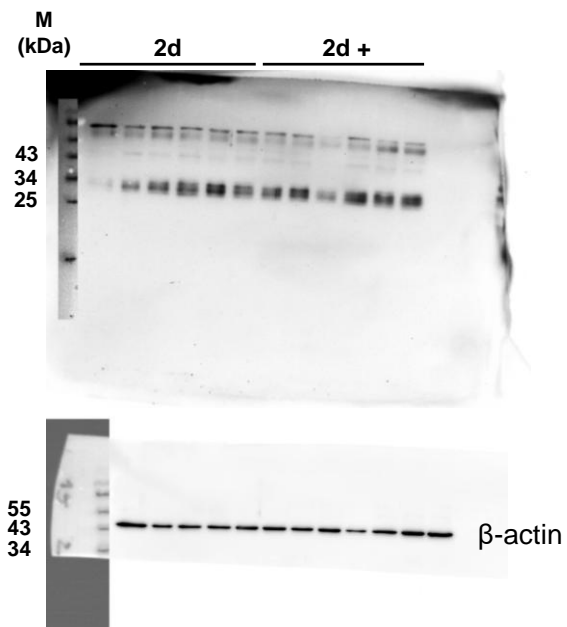

**d**

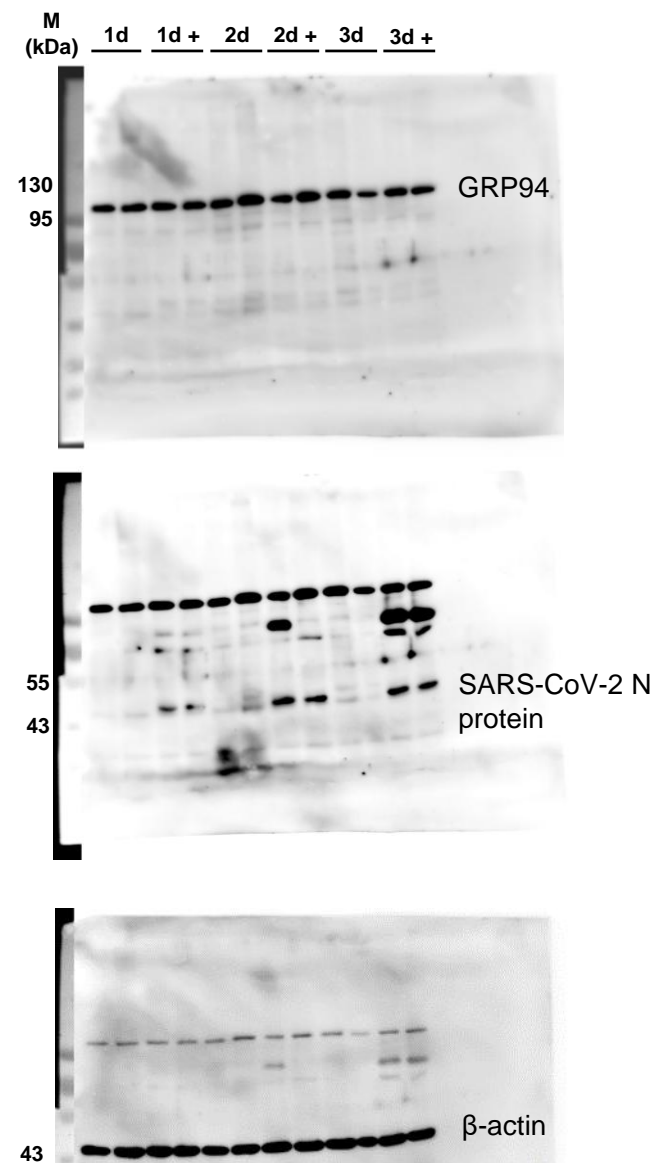

# Figure S7

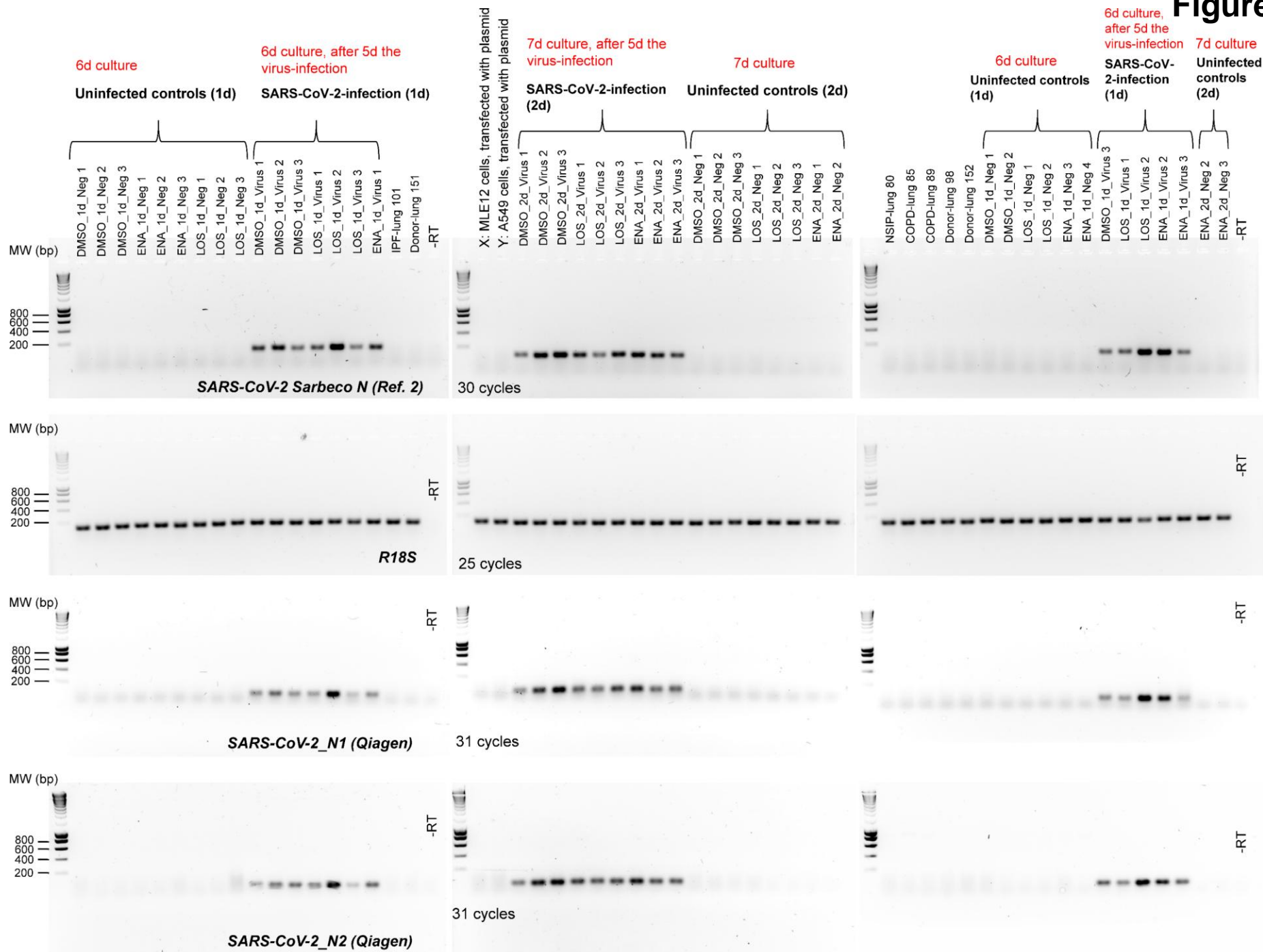

Supplement: Supplementary file 1 — Supplementary Material 1. [file 12931_2025_3463_MOESM1_ESM.zip › Uncropped_Raw Data_Covid_MS_PM.pdf]

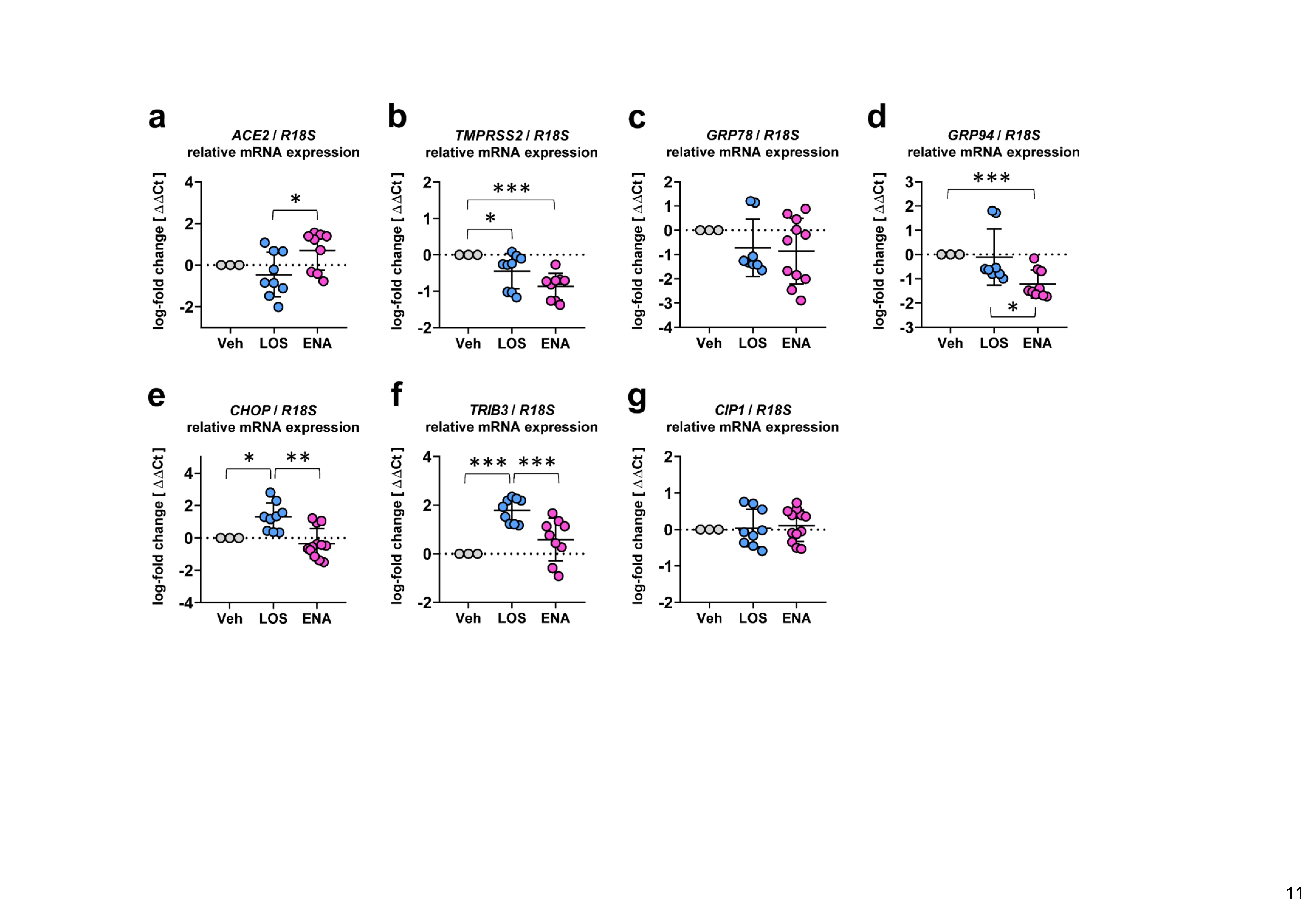

Supplement: Supplementary file 1 — Supplementary Material 1. [file 12931_2025_3463_MOESM1_ESM.zip › Figure S1.tiff]

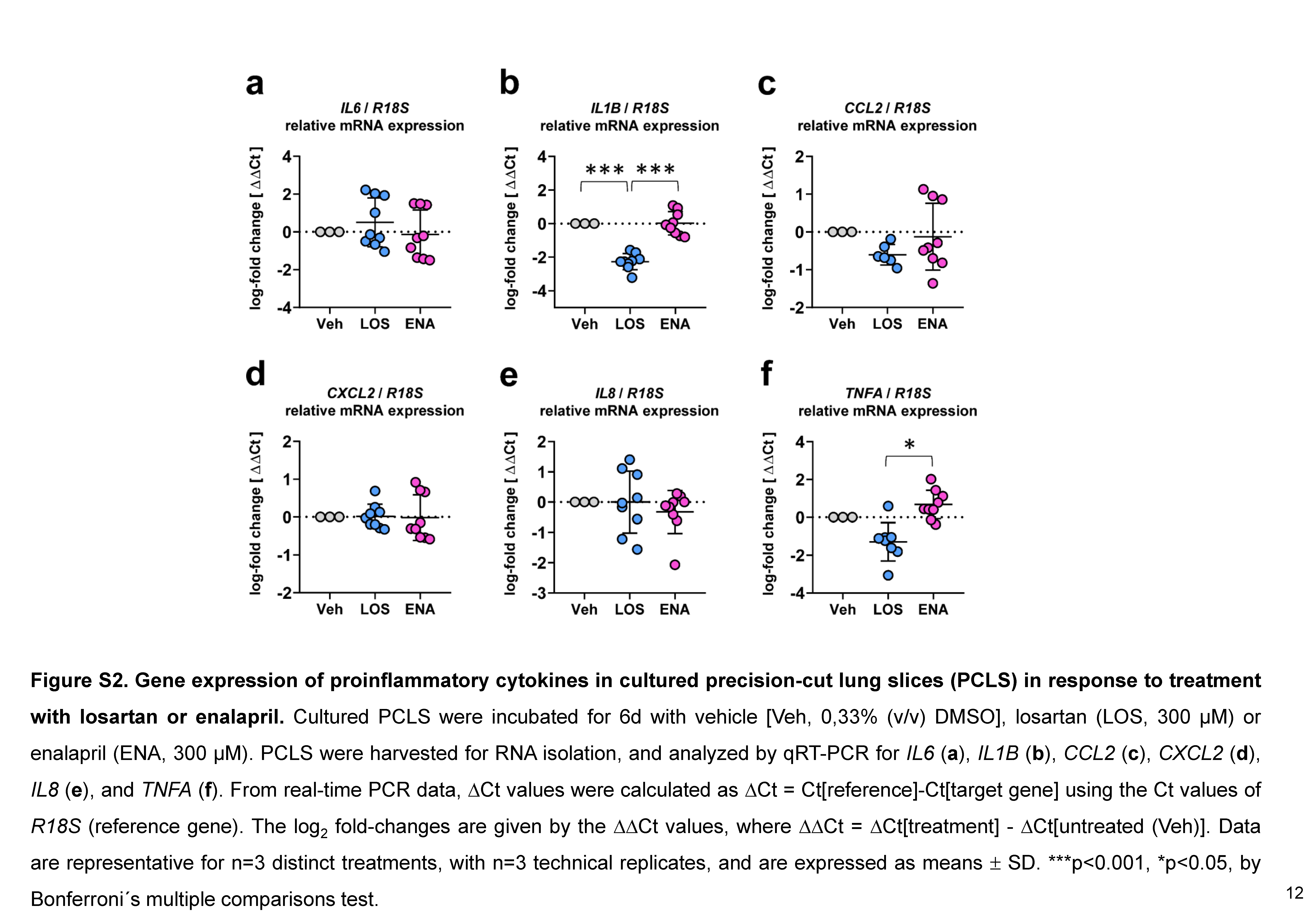

Supplement: Supplementary file 1 — Supplementary Material 1. [file 12931_2025_3463_MOESM1_ESM.zip › Figure S2.tiff]

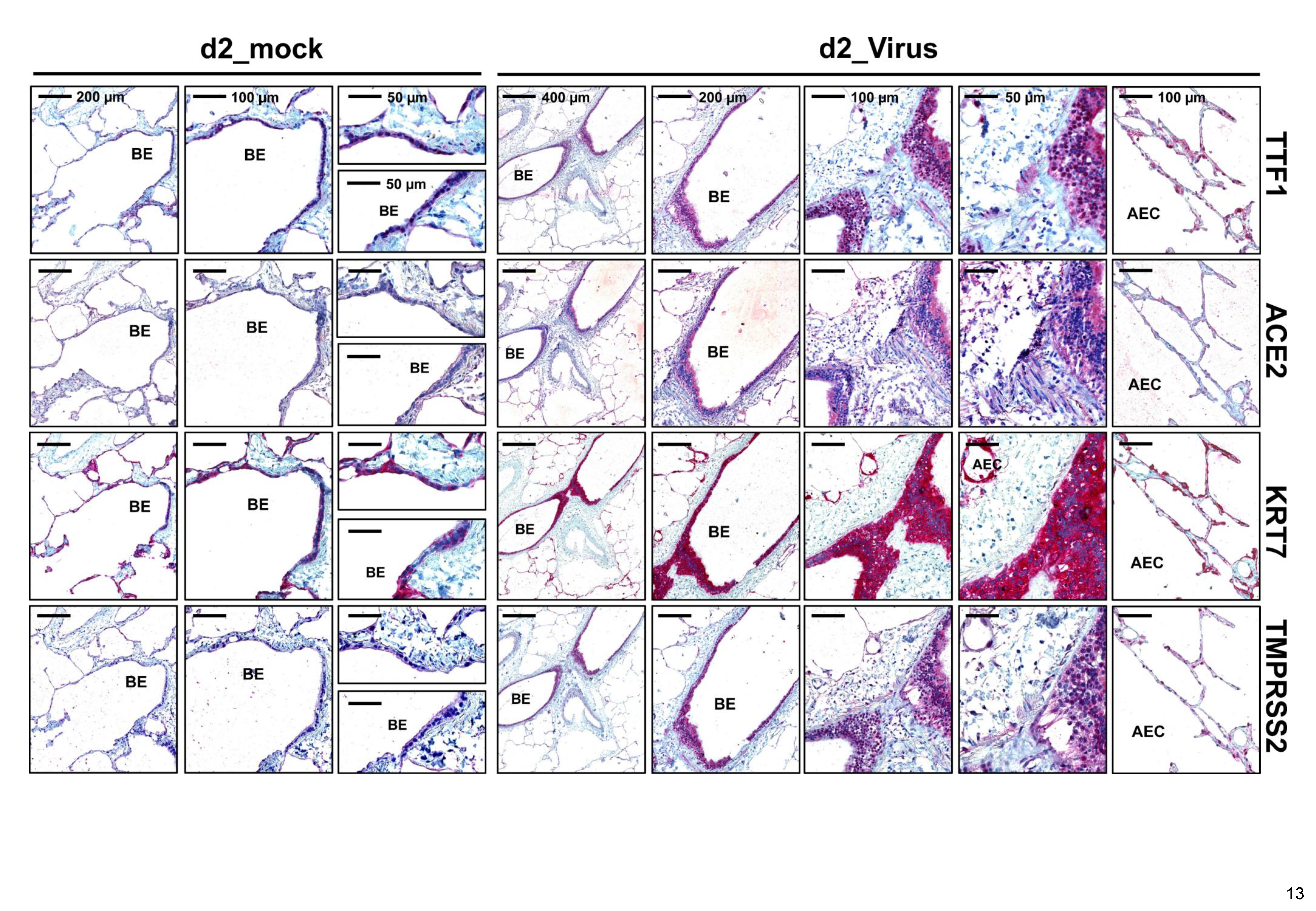

Supplement: Supplementary file 1 — Supplementary Material 1. [file 12931_2025_3463_MOESM1_ESM.zip › Figure S3.tiff]

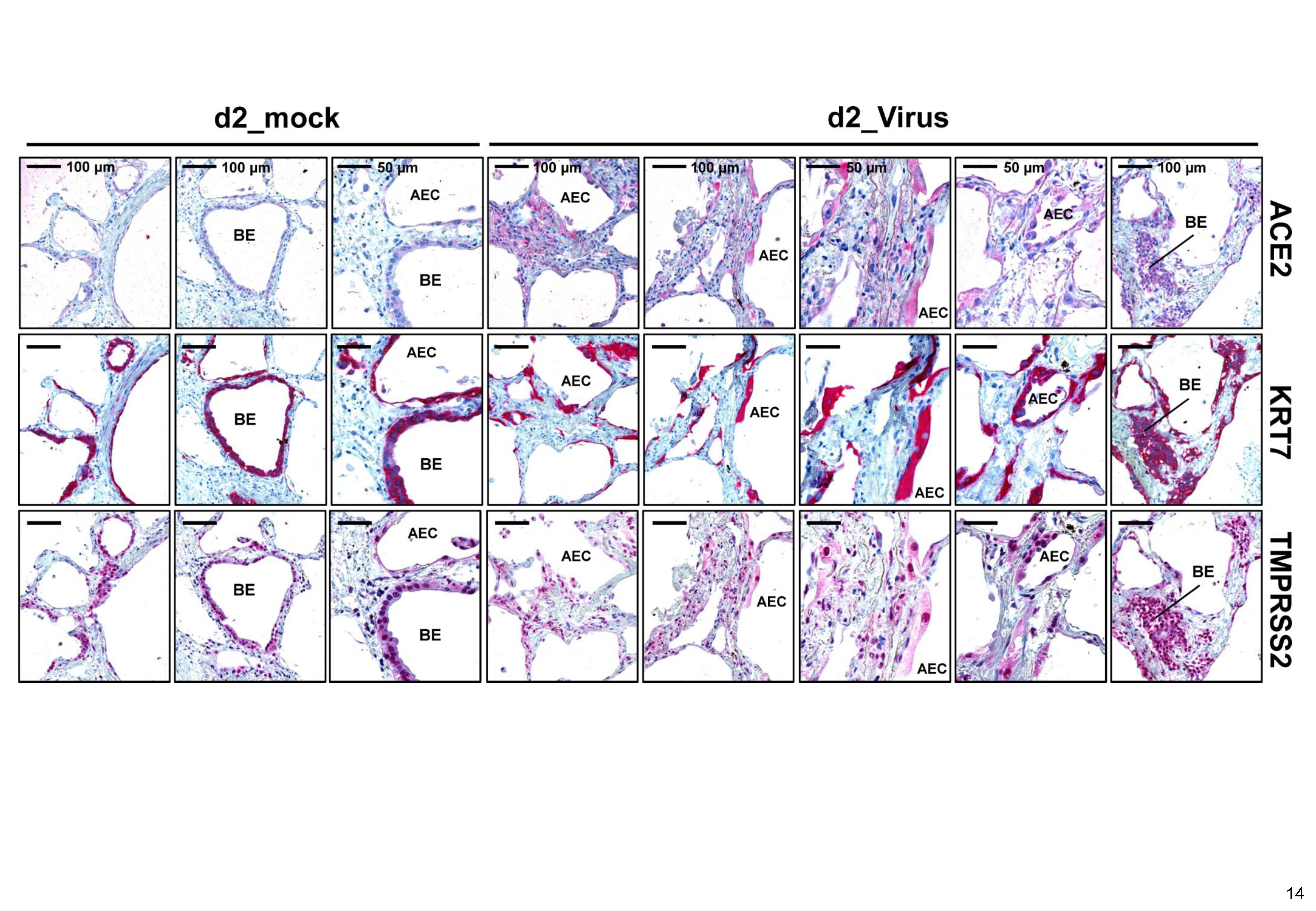

Supplement: Supplementary file 1 — Supplementary Material 1. [file 12931_2025_3463_MOESM1_ESM.zip › Figure S4.tiff]

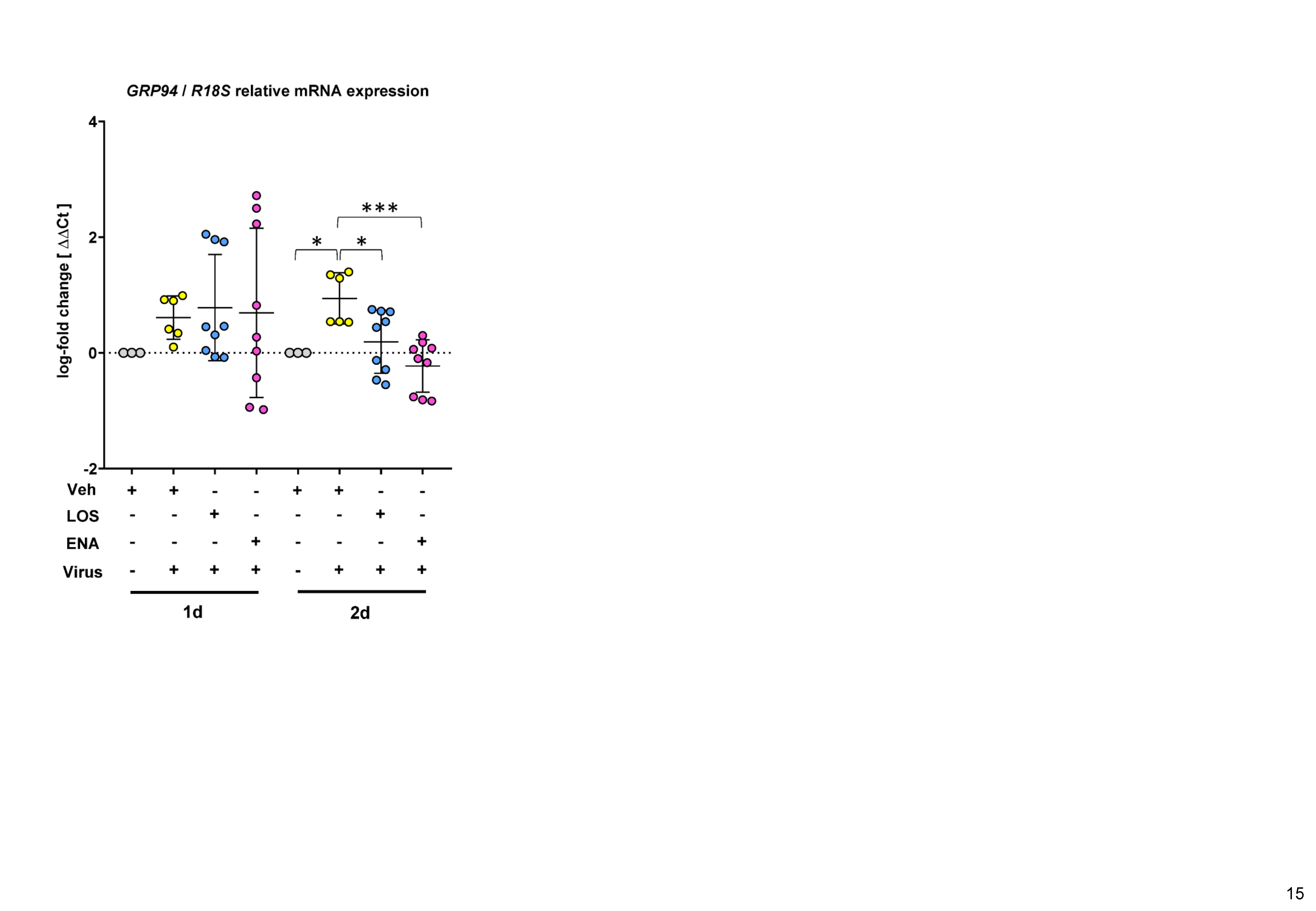

Supplement: Supplementary file 1 — Supplementary Material 1. [file 12931_2025_3463_MOESM1_ESM.zip › Figure S5.tiff]

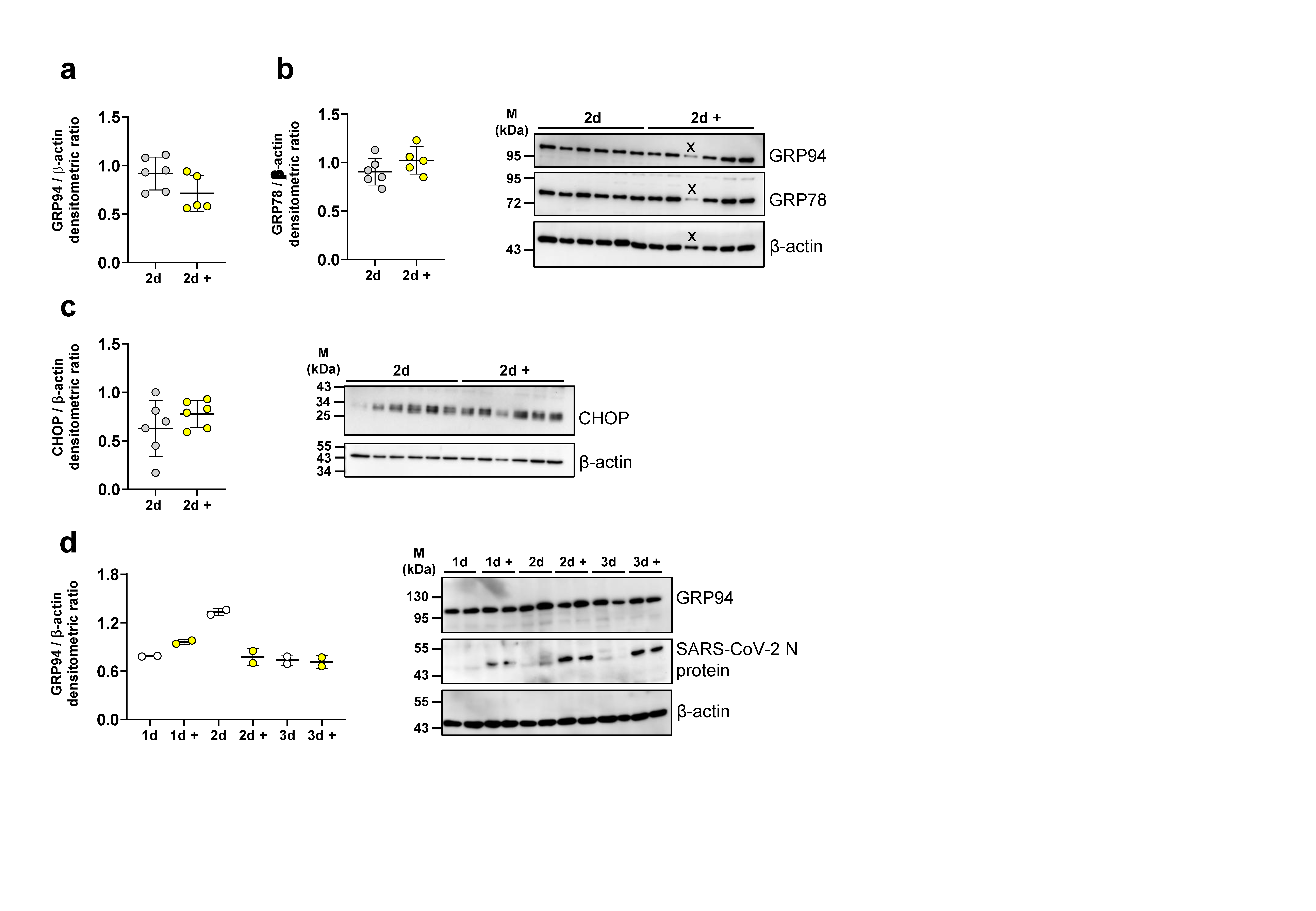

Supplement: Supplementary file 1 — Supplementary Material 1. [file 12931_2025_3463_MOESM1_ESM.zip › Figure S6.tiff]

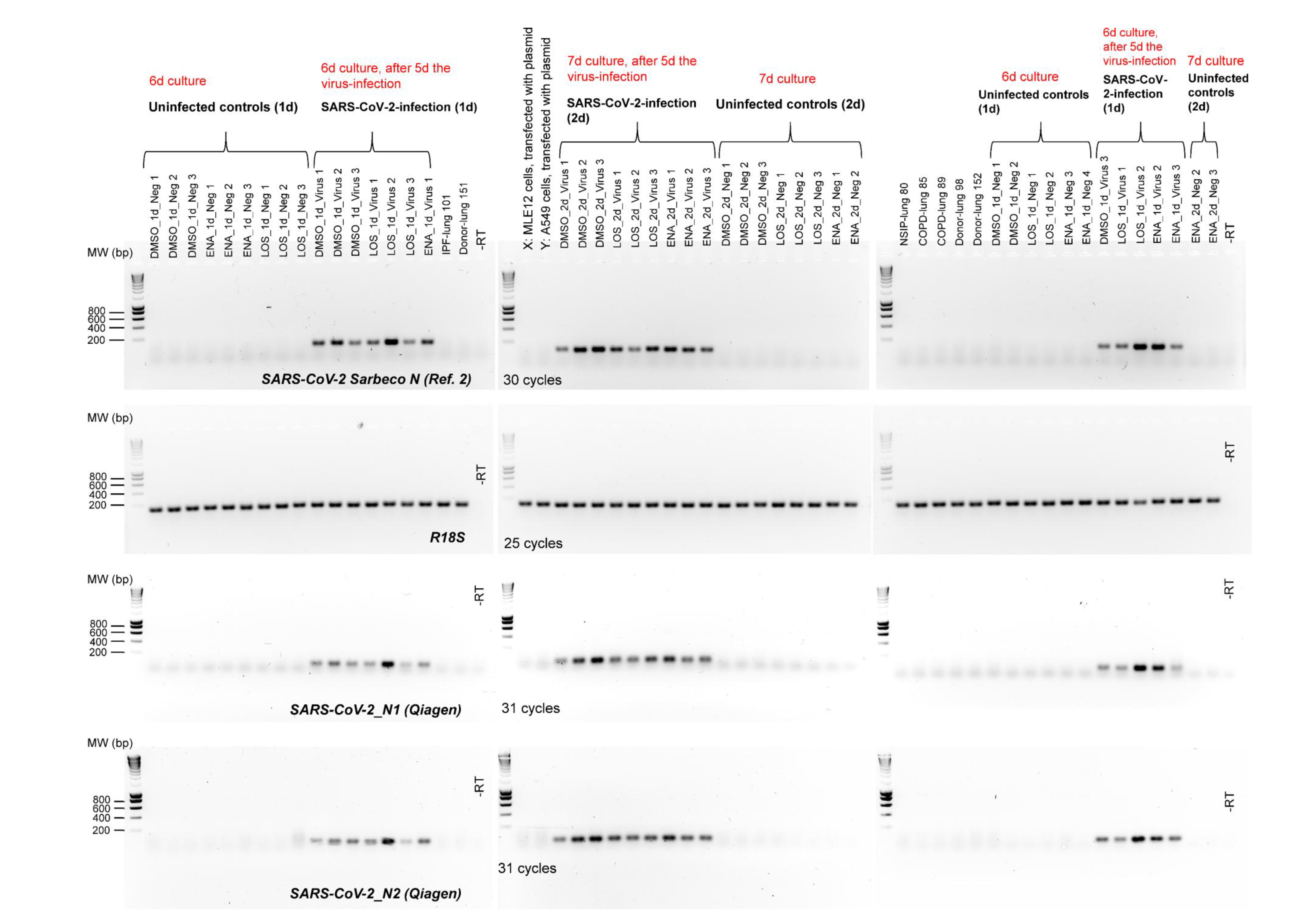

Supplement: Supplementary file 1 — Supplementary Material 1. [file 12931_2025_3463_MOESM1_ESM.zip › Figure S7.tiff]

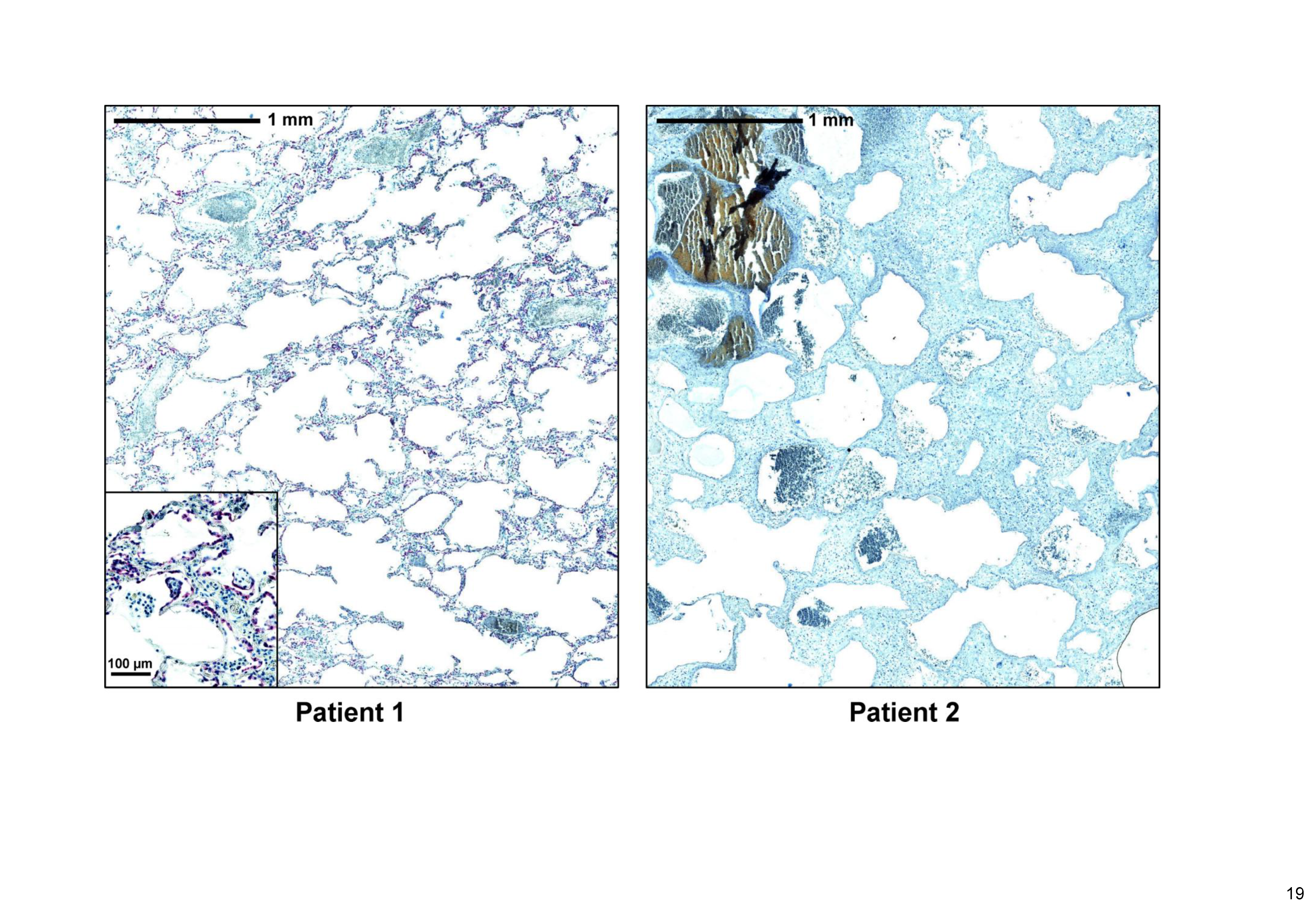

Supplement: Supplementary file 1 — Supplementary Material 1. [file 12931_2025_3463_MOESM1_ESM.zip › Figure S8.tiff]
